# Quantum dots of SnS and SnS_2_ modified with Rhodamine B – sonochemical synthesis, improved sono- and photocatalytic activity, and kinetic modeling

**DOI:** 10.1016/j.ultsonch.2026.107990

**Published:** 2026-08-03

**Authors:** Grzegorz Matyszczak, Albert Yedzikhanau, Tomasz Plocinski, Piotr Dluzewski, Aleksandra Fidler, Cezariusz Jastrzebski, Aleksandra Drzewiecka-Antonik, Anna Wolska, Jakub Zabrzycki, Louis Peraud, Krzysztof Krawczyk

**Affiliations:** a Department of Chemical Technology, Faculty of Chemistry, Warsaw University of Technology, Noakowski Street 3, 00-664 Warsaw, Poland; b Faculty of Materials Science and Engineering, Warsaw University of Technology, Wołoska Street 141A, 02-507 Warsaw, Poland; c Institute of Physics Polish Academy of Sciences, Al. Lotników 32/46, 02-668 Warsaw, Poland; d Faculty of Physics, Warsaw University of Technology, Koszykowa Street 75, 00-662 Warsaw, Poland; e Faculty of Chemical and Process Engineering, Warsaw University of Technology, Ludwika Waryńskiego Street 1, 00-645 Warsaw, Poland

**Keywords:** Tin(IV) sulphide, Tin(II) sulphide, Quantum dots, Sonocatalysis, Photocatalysis, Langmuir-Hinshelwood model

## Abstract

The aim of this study was to sonochemically synthesize SnS and SnS_2_ quantum dots modified *in situ* with organic dye. For this purpose Rhodamine B, a dye from the xanthene group, is used. The resulting nanomaterials were characterized with the following techniques: X-ray powder diffraction, XPS, Raman and EDX spectroscopic techniques to confirm the production of desired compounds. HR-TEM observations prove the formation of quantum dots while the Tauc method was used to study the optical energy bandgap of both modified and unmodified nanomaterials. Raman studies showed not only that nanometric samples of SnS and SnS_2_ were obtained, but the recorded fluorescence from Rhodamine B indicates the presence of its molecules on the surface of these nanoparticles. The modification with Rhodamine B preserved the morphology and phase composition of tin sulphides but caused the occurrence of additional electron transitions as revealed by Tauc analysis. The contact angle measurements showed increase and decrease of contact angle due to modification with dye, for SnS_2_ and SnS respectively. The photo- and sonocatalytic activities of both modified and unmodified quantum dots are studied and compared, including kinetic analyses, investigations of influence of degree of functionalization and catalyst’s amount, and determination of reactive intermediates (scavenging experiments). The modification of SnS with Rhodamine B improved it’s sono- and photocatalytic activity. The investigation of the best catalyst after 5 cycles of sonocatalytic process showed it’s oxidation to SnO_2_. Scavenging experiments revealed that the main reactive intermediate in the case of SnS with optimized amount of dye was radical anion •O_2_^−^. Finally, the n-th order kinetic model provided the most accurate description of photocatalytic processes.

## 1 Introduction

The beginning of application of ultrasound for conduction of chemical reactions, which is called sonochemistry, is dated for year 1927 when Wood, Loomis and Richards reported their first discoveries regarding biological and physical effects of ultrasound, as well as the observed acceleration of chemical reaction due to the action of ultrasound [1], [2], [3]. Since then the application of ultrasound spread to various branches of chemistry, including analytical chemistry (sample preparation, mass transfer improvement in electroanalytical chemistry) [4], [5], [6], organic chemistry (speeding up the reactions and possibly changing their mechanisms) [7], [8], [9], [10], polymer chemistry (speeding up of polymerization reactions and affecting properties such as molecular weight) [11], food chemistry (e.g. improving extraction processes) [12], [13], [14], and inorganic chemistry (synthesis of metallic and semiconducting nanomaterials) [15], [16], [17], [18]. The most important phenomena underlying the sonochemical effect is the acoustic cavitation [19]. Ultrasonic wave, while passing through the liquid medium, causes fluctuations of pressure. The temporal drop in pressure may decrease the solubility of gases dissolved in the medium and allow the formation of acoustic bubbles [19]. However, the tensile strength present in the medium typically makes this formation impossible [19]. The observed formation of acoustic bubbles is in fact caused by discontinuities in the medium, such as micro-gaps filled with gas, and micro-bubbles [19]. The acoustic bubble, after being formed, undergoes several cycles of rarefaction and compression until it reaches its resonant, maximum size when it collapses generating extreme conditions – at this moment the temperature reaches thousands of Kelvins and the pressure achieves ten thousands of standard atmospheres inside the bubble [16], [19]. Under such conditions many chemical reactions may occur, including − possibly the most basic of sonochemical reactions − water sonolysis [16], [19]:

H_2_O → H**·** + **·**OH (1).

The application of ultrasound in chemistry includes especially the synthesis of inorganic nanomaterials [15], [16], [17], [18]. This approach meets the criteria of Green Chemistry as it allows to use less toxic solvents, such as water, instead of toxic and high-boiling solvents typically used in methods of nanoparticles preparation such as the solvothermal or hot-injection methods [16], [20]. The ultrasound may cause not only the chemical effects but also physical ones such as agglomeration and melting of metallic particles due to the increase in their velocity before and during collisions [20]. Sonochemistry was successfully applied in the synthesis of various simple (e.g. CdS, SnS, SnS_2_) and complex (e.g. AgInS_2_, CuInS_2_, Cu_2_ZnSnS_4_) inorganic compounds [17], [21], [22], [23], [24]. In the context of sonochemical synthesis of tin sulphides, SnS and SnS_2_, it was shown that the properties of product, such as crystallite size, morphology, and optical energy bandgap, soundly depend on synthetic conditions including sonication time, type of used solvent, and ratio of reagents [25].

Mentioned SnS and SnS_2_ compounds are interesting materials that are investigated for many applications. Tin(IV) sulphide is a n-type semiconductor which energy band gap for bulk phase is c.a. 2.4 eV [26], while tin(II) sulphide exhibits p-type conductivity and energy band gap for a bulk phase of c.a. 1.3 eV [27]. Both compounds also typically adopts different crystal structures – SnS_2_ crystallizes mostly in hexagonal crystal system, while SnS crystallizes in cubic, and orthorombic crystal systems [28], [29]. SnS and SnS_2_ may form other, so-called mixed-valence, tin sulphides such as Sn_2_S_3_ and Sn_3_S_4_ which are mixtures of SnS and SnS_2_ in certain ratios – 1:1 for Sn_2_S_3_ and 2:1 for Sn_3_S_4_ [30], [31], [32]. Novel mixed-valence tin sulphides, so far not obtained experimentally, including Sn_3_S_5_, Sn_4_S_5_, and Sn_4_S_7_ were theoretically investigated for applications in solar cells [33]. A survey of most recent applications (last two years) of tin sulphides shows their potential in solar cells, photocatalysis, optoelectronics, supercapacitors, fuel cells, and exosome detection [34], [35], [36], [37], [38], [39], [40], [41]. This shows that studies of well-known SnS and SnS_2_ compounds are still actively developing.

Nevertheless, some characteristics of tin sulphides and other materials are not sufficient to support their application. Useful properties of nanomaterials may be further enhanced by modifications with organic ligands, for example bandgap may be tuned by functionalization with conjugated organic groups and chromophores [42], [43], [44], [45], [46]. Such modification was achieved, for example, for nanopowders of kesterite Cu_2_ZnSnS_4_ prepared in high-temperature reaction [46]. In the sonochemical synthesis, modification of SnS_2_ nanopowder was achieved using commercial dye Phenol Red what improved it’s photocatalytic activity [47]. Modifications of SnS made so far were performed with dodecylamine, trioctylphosphine, hexamethyldisilazane, and oleic acid, while SnS_2_ was modified only with mentioned dye Phenol Red [47], [48], [49], [50].

This study presents an approach for *in situ* modification of SnS and SnS_2_ quantum dots in sonochemical synthesis using commercial dye Rhodamine B from xanthene dyes group. Such type of organic compounds wasn’t used for modification of SnS and SnS_2_ quantum dots so far. Rhodamine B was used for the modification of graphene, CdS, and CdSe quantum dots affecting charge transfer and possibly catalytic activity [51], [52], [53]. The influence of modification on photo- and sonocatalytic activity, as well as on properties of synthesized quantum dots, is studied. Obtained nanomaterials are characterized with techniques such as powder X-ray diffraction, scanning electron microscopy, energy-dispersive X-ray spectroscopy, X-ray photoelectron spectroscopy, Raman spectroscopy, Tauc method, high-resolution transmission electron microscopy, Mott-Schottky analysis and contact angle measurements. Data gathered from catalytic experiments are modeled with various kinetic models to compare their usability. The mechanisms of catalytic processes, with respect to the reactive intermediates, are also studied.

## 2 Materials and methods

### 2.1 Materials and reagents

**Fig. 1:**
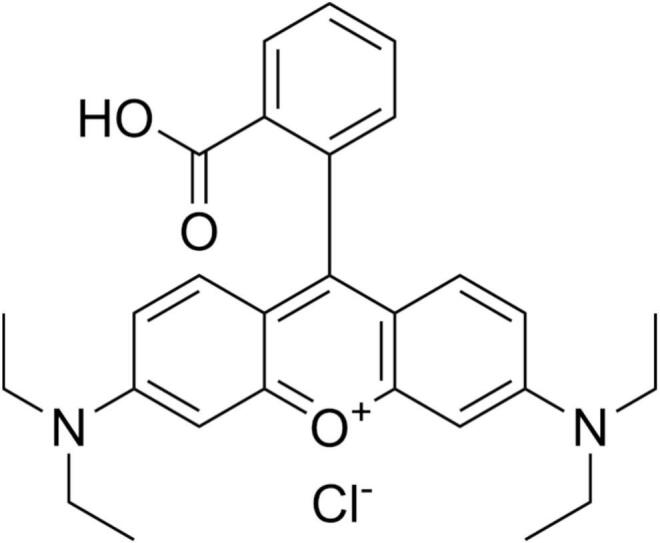
Structural formula of dye Rhodamine B.

All chemicals used in this study were of analytical grade. In sonochemical syntheses, SnCl_4_·5H_2_O and SnCl_2_·2H_2_O were used as tin sources while thioacetamide (TAA) was used as a sulfur source. Rhodamine B dye was used as organic ligand (see Fig. 1 for it’s molecular structure). Distilled water was used as a solvent and ethanol was used for the purification of prepared suspensions.

### 2.2 Sonochemical syntheses and purification of products

The procedure for syntheses and purification of obtained powders was adopted from previous Matyszczak et. al. investigation [37].

The sonochemical syntheses were conducted using 50 mL conical flasks. An ultrasonic cleaner (PS 10A) was used as the indirect ultrasound source. It generates an ultrasound of 40 kHz frequency with nominal power of 60 W. The acoustic power of utilized cleaner was determined using calorimetric method yielding value 27.9 W/L.

In all syntheses following amounts of reagents were used: 701 mg of SnCl_4_·5H_2_O (synthesis of SnS_2_ quantum dots) or 452 mg of SnCl_2_·2H_2_O (synthesis of SnS quantum dots), 376 mg of thioacetamide (in both cases), and 20 ml of distilled water as solvent (also in both cases). All reagents were stirred for 10 min with a magnetic stirrer before the start of the reaction. The sonication lasted 120 min. Immediately after the reaction (sonication) the conical flasks were opened and kept under laboratory hood for 10 min to remove the toxic gases possibly generated in the process.

Centrifugation was used to separate resulting nanopowders from the reaction mixture. After the first centrifugation, a supernatant (reaction mixture) was removed and a portion of fresh ethanol was added. Then the powder was suspended by sonication (10 min) and mixed by plastic Pasteur pipette. Then the centrifugation was repeated, supernatant was removed again, and another portion of fresh ethanol was added. After that, powder was again suspended. Then the suspension was centrifuged, supernatant was removed and the powder was finally suspended in another portion of fresh ethanol.

### 2.3 UV–Vis spectrophotometry (Tauc method)

The UV–Vis spectra were collected with UV1600 spectrophotometer (AOE Instruments) and then was used to perform analysis based on the Tauc method as described in our previous study [37]. Spectra were measured using ethanol suspensions of nanopowders produced in syntheses.

### 2.4 Raman spectroscopy

A Renishaw InVia Reflex spectrometer was used to perform Raman measurements. The measurements utilized a backscattering configuration with a Leica microscope module. A 50x short-range microscope objective was used to excite the sample and accumulate the scattered radiation. Three laser lines were used as excitation in the various measurements: 1064 nm, 514 nm, and 633 nm from Nd YAG, Ar ion, and He-Ne lasers, respectively. For the 1064 nm laser line, a diffraction grating of 830 g/mm was used in the detection path, while for the 514 and 633 nm lines, a grating of 1800 g/mm was used. The laser beam power was adjusted to avoid structural changes and thermal effects in the samples.

### 2.5 XPS investigations

The nanodots samples for the XPS measurements were prepared accordingly to the method described in the previous publication [37], i.e. suspensions of samples were dripped on Cu conductive tape and left to dry in laboratory fume cupboard until visible sample layer was obtained. X-ray photoelectron spectra (XPS) were recorded by Prevac set-up equipped with high intensity monochromatic X-ray Al Kα (1486.69 eV) source Scienta MX 650 (set at 150 W), Scienta R4000 hemispherical analyser. Charge neutralization was used only in case of the powder Rhodamine B sample. The narrow scans were acquired with pass energy 200 eV and the step 0.2 eV. The full width at half maximum (FWHM) for the Au 4f 7/2 line measured at the same experimental condition was equal to 0.6 eV. The energy scale was calibrated setting the C 1 s line at the position 284.8 eV. Spectra were analysed using the commercial CASA XPS software package (Casa Software Ltd, version 2.3.17) with Tougaard background and a GL (30) line shape (70 % Gaussian, 30 % Lorentzian).

### 2.6 SEM and EDX investigations

The Scanning Electron Microscopy SEM investigations were performed on the microscope made by Hitachi High Technologies model S5500. The powder samples were deposited on carbon tape after drying. The observations were taken at 30 keV accelerating voltage. The Secondary Electrons SE were used to register images of surface topography at magnification range up to 500 000 times. This microscope has unique construction, where the sample is introduced in the pole piece of objective lens on the side entry holder. The same concept is used in Transmission Electron Microscope. This special conditions can determine the ultimate resolution below 1 nm.

### 2.7 Powder X-ray diffraction investigations

The powder diffraction data were collected for dried samples using X-ray diffractometer Bruker D8 ADVANCE and filtered Cu Kα radiation (λ = 0.154056 nm) at room temperature. The parameters during measurements were following: voltage − 40 kV, current – 40 mA, angle range from 10° to 80°, step Δ2Θ − 0.025°, counting time – 3 s.

### 2.8 HR-TEM investigations

Specimens for TEM investigations were prepared as follows – 0.5 mg of the powdered material was suspended in ethanol (3 ml) by sonicating for 5 min in an ultrasonic bath. Then a 30 μl of the suspension was deposited on a standard TEM copper grid coated with 30 nm thick carbon film. The grid was subsequently left under reduced pressure to evaporate the alcohol.

In order to evaluate the morphology, crystal structure and elemental composition of the specimens they were subjected to a Transmission Electron Microscopy (TEM) investigations. The observations were carried out using FEI Titan Cubed 80–300 microscope operated at 300 kV in bright-field TEM mode. Both overview and high-resolution images were collected and recorded using Gatan BM-Ultrascan CCD camera. The elemental composition was determined by Energy Dispersive X-ray Spectroscopy (EDS) with the use of EDAX 30 mm^2^ Si (Li) detector, having a collection angle of 0.13 srad.

### 2.9 Mott-Schottky analyses

The Mott-Schottky analyses were performed to confirm the semiconductors’ conductivity type and estimate their flat-band potentials (E_FB_). To do this, electrochemical inks were prepared by sonicating 0.5 mg of each sample in 250 µl ethanol for 30 min. Subsequently, 3.50 µl of each ink was placed on the glassy carbon electrode with a diameter of 3 mm. The so-prepared working electrodes were then dried at 50℃ for three hours. Measurements were conducted in the dark in a 0.1 M KCl aqueous solution and using a Bio-Logic SAS SP-200 potentiostat. The KCl solution was purged with argon for 15 min before every measurement. The three-electrode system was used, with an Ag/AgCl electrode (saturated with a 3-mole NaCl solution) and a platinum wire as the reference and counter electrodes, respectively. Measurements were conducted at room temperature using an AC amplitude of 10 mV over the frequency range 100 kHz–10 Hz, following the methodology described in the literature [54].

### 2.10 Photo- and sonocatalytic experiments

The procedures for sono- and photocatalytic experiments were adopted from our previous study [37].

**Fig. 2:**
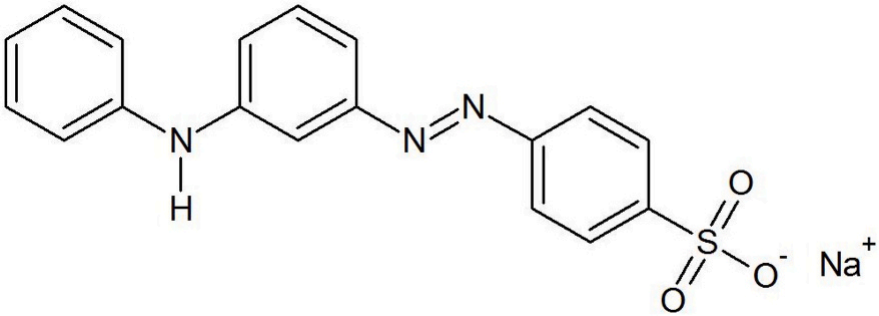
Structural formula of azo-dye Metanil Yellow.

The degradation experiments were performed using model pollutant azo-dye Metanil Yellow which structural formula is displayed in Fig. 2. It’s highly toxic and, as an azo-dye, may exhibit mutagenic and carcinogenic actions against human [55], [56], [57]. It is illegally used in food industry in India, e.g. as adulterant in turmeric powders [58], [59].

A certain portion of catalyst (powder of dried suspension produced in sonochemical synthesis) was weighed and placed in 5 mL of distilled water and sonicated for 40 min. During this period the suspension was additionally stirred with plastic Pasteur pipette. Next, the resulting suspension was placed in beaker containing 30 mL of dye solution and equipped with a magnetic bar. To ensure the quantitative transfer of catalyst to the dye solution, the leftovers of catalysts suspension were washed 5 times with 1 mL of distilled water (each time additionally applying sonication for 10 s). The final volume of mixture in the beaker was 40 mL. Each of above mentioned volumes was measured with the graduated pipette. The prepared mixture was then stirred under dark conditions on magnetic stirrer with speed of 800 rpm for 30 min to ensure the adsorption–desorption equilibrium. After that, the mixture was used in degradation experiments.

The photocatalytic process was conducted in the same glass beaker at room conditions. As a source of UV radiation an UV-C lamp of nominal power 72 W was used. In each photocatalytic experiment the position of beaker in reference to the UV lamp, as well as the shape and size of beaker, was exactly the same. The experiment (i.e. irradiation) lasted 120 min each time. Experiments were conducted under dark conditions.

The sonodegradation experiments were conducted in centrifuge tubes (falcons) of 14 mL volume. For each experiment a portion of 7 mL of prepared catalysts suspension in dye solution was measured with graduated pipette and placed in each falcon. Four falcons were then symmetrically placed in ultrasonic bath and sonicated for 120 min under dark conditions. The source of ultrasound was the same as in the case of sonochemical syntheses. The positions of falcons in ultrasonic bath were precisely controlled and kept the same in each sonocatalytic experiment. The level of water in bath was equal to the level of liquids in falcons.

After both kind of processes the mixture was centrifuged twice at a speed of 8000 rpm for 8 min to obtain clear dye solution. The absorbance was measured at wavelength of 440 nm using UV–Vis spectrophotometer (model UV1600, AOE Instruments). Chosen value of wavelength corresponds to the maximum of absorbance of aqueous solution of Metanil Yellow. Each experiment, including blind test, was conducted 4 times.

The color removals during the sono- and photocatalytic processes were calculated using the following equation:

(2) $$\mathrm{CR}=\frac{A_{O}-A_{E}}{A_{O}}\cdot 100\text{\%}$$

Where:

CR- percentage color removal [%].

$A_{0}$- absorbance of dye solution before process [-].

$A_{E}$- absorbance of dye solution after process [-].

### 2.11 Contact angle measurements

The measurements of contact angle were performed on thick layers of investigated materials prepared on microscope slide. Layers were prepared by dripping of ethanol suspension of corresponding nanomaterial and then drying. The contact angle was measured after placing the drop of relevant solvent on the layer. For calculation of the mean contact angle 6 results were taken (for 3 drops, for each of drop's two sides). Three solvents were used: distilled water, diiodomethane CH_2_I_2_, and glicerine.

### 2.12 Scavenging experiments

To perform the scavenging experiments an relevant scavenger (KI, ascorbic acid, *tert*-buthanol) was added to the reaction mixture before the start of the stabilization of the adsorption–desorption equilibrium. Potassium iodide and ascorbic acid were added in such amount that their final concentration in the reaction mixture was 10 mM. Tert-buthanol was added in an amount of 1 mL – at the same time the amount of water in the reaction mixture was decreased by 1 mL. Two samples were monitored spectrophotometrically – after stabilization of the adsorption–desorption equilibrium (30 min of stirring under dark conditions) and after 120 min of the corresponding catalytic reaction.

### 2.13 Kinetic modeling

The mean values of concentrations of degraded dye at certain moments of time during both sono- and photocatalytic processes were taken as the data for the kinetic modeling. Three different kinetic models were used to fit the experimental data:

1 the pseudo-nth order kinetic model (n > 1):

(3) $${c\left(t_{i}\right)}^{\left(1-n\right)}={c\left(t_{0}\right)}^{\left(1-n\right)}-\left(1-n\right)kt_{i}$$

2 the pseudo-first order kinetic model:

(4) $$c\left(t_{i}\right)=c\left(t_{0}\right)∙e^{-{\mathrm{kt}}_{i}}$$

3 the Langmuir-Hinshelwood kinetic model:

(5) $$\left(c\left(t_{i}\right)-c\left(t_{0}\right)\right)+K^{\prime }ln\left(\frac{c\left(t_{i}\right)}{c\left(t_{0}\right)}\right)=-k_{2}c_{K}t_{i}$$

A differential evolution algorithm, as implemented in scipy Python library, was used to fit the above models to the experimental data, using the following values of parameters: maxiter = 1000, tolerance = 10^-6^, population size = 200. The target function was a sum of squared errors (SSE):

(6) $$\mathrm{SSE}=\sum_{i=1}^{n}{\left({c\left(t_{i}\right)}_{\exp}-{c\left(t_{i}\right)}_{\mathrm{fitted}}\right)}^{2}$$

where:

c(t_i_)_exp_ – experimental dye concentration at time t_i_.

c(t_i_)_fitted_ – predicted dye concentration at time t_i_.

n – number of experimental points.

However, the mean absolute percentage error (MAPE) was used to directly compare the fitness of various models as the percentage error is easier to percept than SSE:

(7) $$\mathrm{MAPE}=\frac{\sum_{i=1}^{n}\frac{\left|{c\left(t_{i}\right)}_{\exp}-{c\left(t_{i}\right)}_{\mathrm{fitted}}\right|}{{c\left(t_{i}\right)}_{\exp}}}{n}$$

## 3 Results and Discussion

### 3.1 Visual investigation of products of syntheses and the result of modification

The nanopowders prepared without the addition of Rhodamine B were brown-orange (for SnS_2_) or dark brown (for SnS) [37]. The suspensions resulting from syntheses performed with the addition of Rhodamine B were violet in the case of syntheses of SnS_2_ quantum dots, and dark brown in the case of syntheses of SnS quantum dots. The supernatants at the final step of purification of both products were clear indicating that – at least in the case of SnS_2_ nanopowders – the modification was successful.

### 3.2 X-ray powder diffraction investigations

**Fig. 3:**
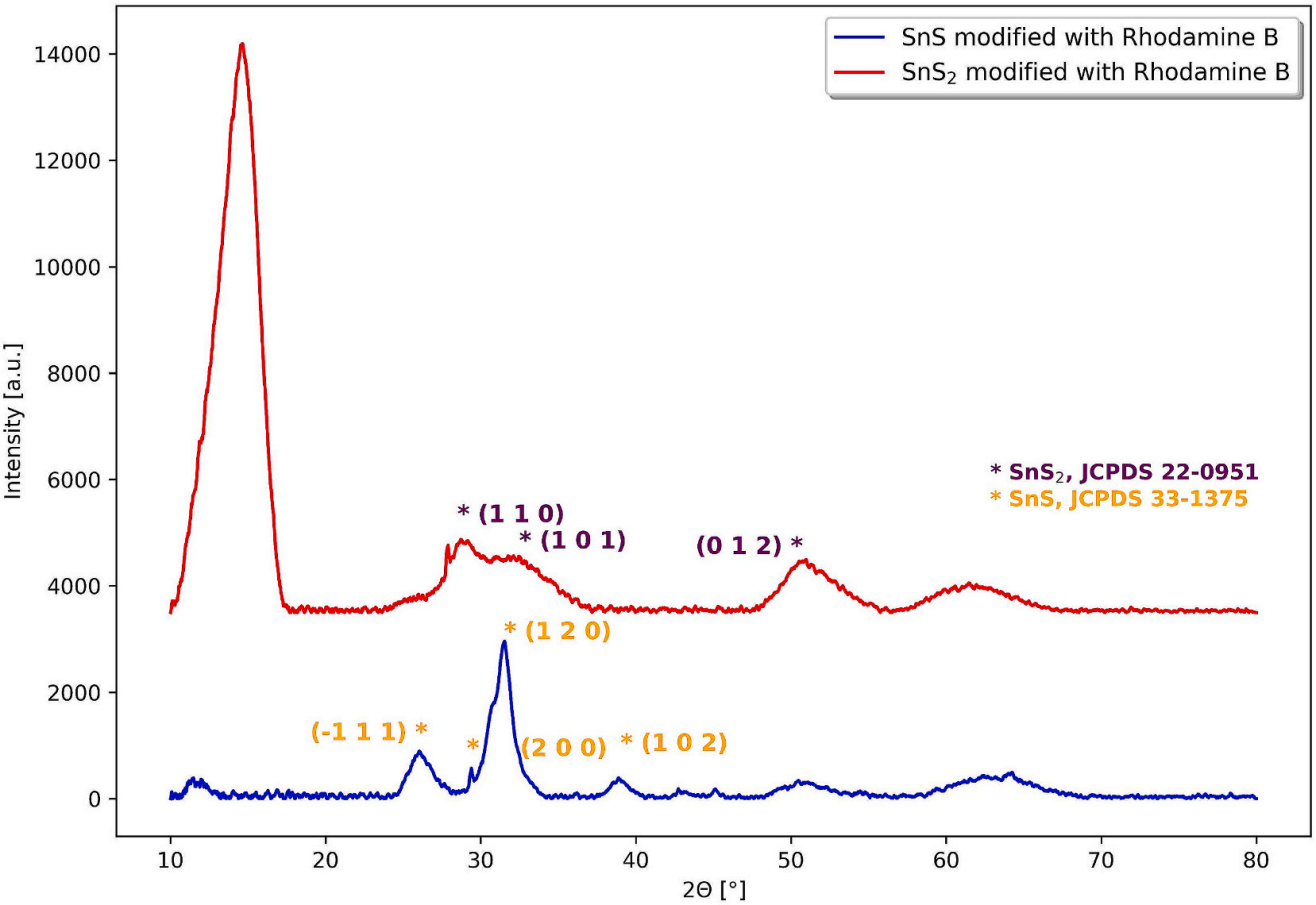
Powder X-ray diffractograms of sonochemically synthesized SnS and SnS_2_ quantum dots modified with Rhodamine B.

The X-ray powder diffraction investigations revealed the presence of nanocrystalline SnS and nanoamorphous SnS_2_ in the prepared samples (Fig. 3). It should be noted that the collected diffractograms are similar to the ones for unmodified SnS and SnS_2_ quantum dots presented previously in the literature [37]. Nanoamorphous character of tin(IV) sulphide nanopowder is indicated by large and wide peak at c.a. 13-14°. There is no evidence for traces of dye in dried, prepared samples according to powder diffractograms. In general, the crystallinity of products is relatively low as many reflexes for SnS and SnS_2_ phases are missing. This fact justifies the need for further characterizations to prove the existence of SnS and SnS_2_ compounds in the products of syntheses.

### 3.3 Raman spectroscopy investigations

**Fig. 4:**
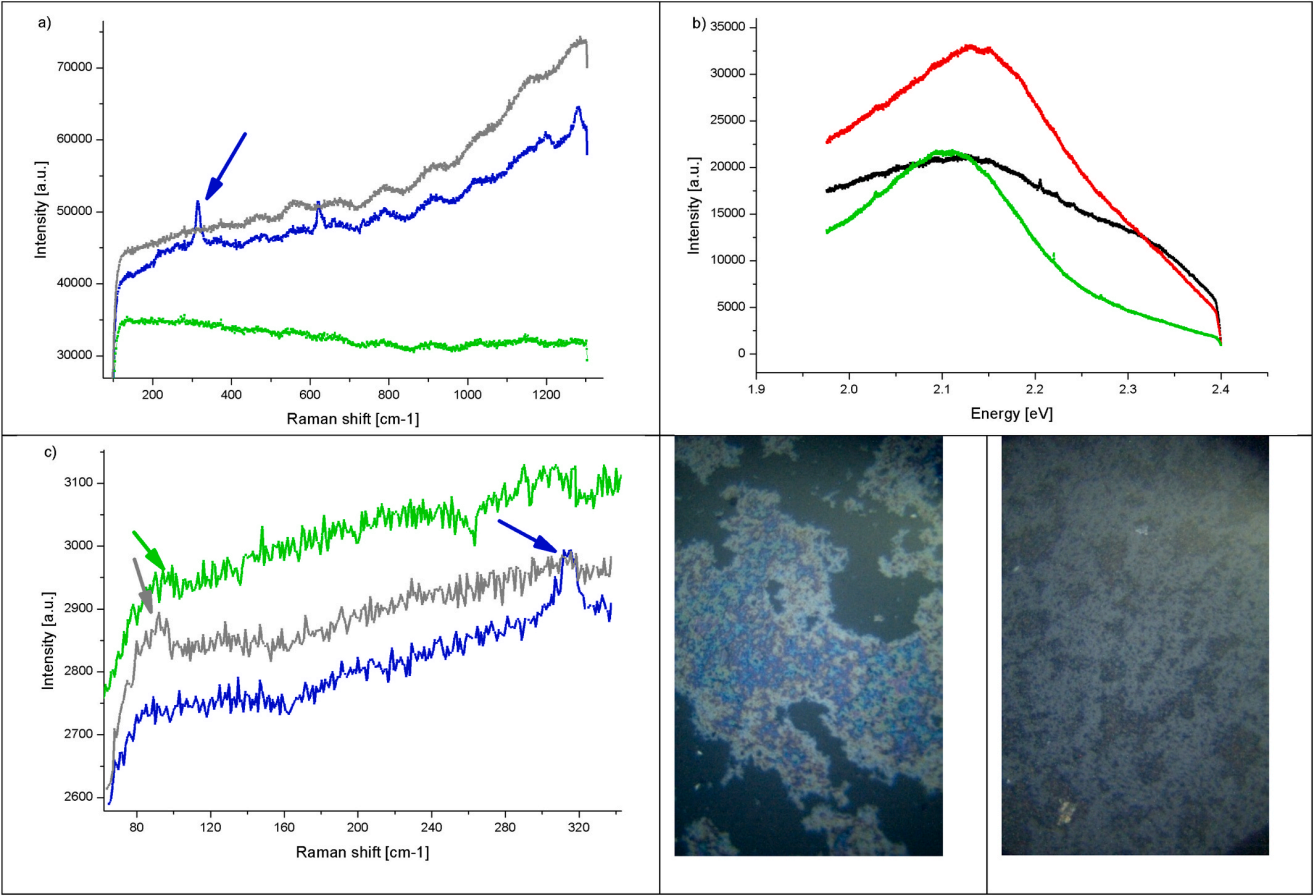
Raman spectra of SnS and SnS_2_ nanograins at excitation at 633 nm (a) and 1064 nm (c), respectively. The spectrum for SnS is marked in dark gray and for SnS_2_ in blue. Arrows indicate characteristic Raman peaks for these crystalline phases. Fluorescence of Rhodamine B molecules [62] (b) present on SnS (red) and SnS_2_ (black) samples. Green color in subfigures a), b), c) indicates experimental data for SnS nanograins after the 5 cycles of sonocatalytic process. The lower right corner shows microscope images using a 50x objective lens for a colored SnS_2_ sample and a gray SnS sample dispersed on a Si wafer substrate.

Raman studies were performed to confirm the crystalline phases of SnS and SnS_2_ in the studied samples. The SnS_2_ crystalline phase has a CdI_2_ structure and P-3 m1 space group symmetry (no. 164). It exhibits a characteristic, very intense Raman peak with A_g_ symmetry at 315 cm^−1^, regardless of excitation [60]. Therefore, detection of this crystalline phase is relatively straightforward. At both 633 nm and 1064 nm excitation, a peak around 315 cm^−1^ is observed, indicating the SnS_2_ phase (Fig. 4 a, c). Only at the 514 nm excitation line, the very strong fluorescence of Rhodamine B completely obscure the Raman spectrum of SnS_2_ (Fig. 4 b).

The situation is different for SnS, which has an orthorhombic structure similar to NaCl and Pnma symmetry (no. 62). SnS nanograin samples are characterized by very low Raman scattering intensity. Therefore, the Raman spectra of nanograins are very difficult to observe, especially when accompanied by very strong fluorescence. Typically, Raman scattering reveals two characteristic Raman peaks with A_g_ symmetry at approximately 192 and 220 cm^−1^ [61]. These studies exploited the fact that, when excited in the near-infrared range, resonant Raman scattering is observed, resulting from the SnS energy gap of approximately 1 eV. The A_g_ symmetry peak located at approximately 96 cm^−1^ becomes dominant.

For 1064 nm excitation, we see a very weak peak at approximately 96 cm^−1^, indicating the SnS structure (dark gray arrow in Fig. 4 c). For samples with the SnS_2_ structure, we see a low-intensity A_g_ peak at approximately 315 cm^−1^ (blue arrow in Fig. 4 c). Resonance Raman scattering is a very good example of confirming the SnS structure in nanograins. It should also be noted that the presence of Rhodamine B fluorescence in the study of SnS and SnS_2_ nanograins (Fig. 4 b) indicates that the surface of these grains has attached the Rhodamine B molecules.

### 3.4 Scanning electron microscopy and energy-dispersive X-ray spectroscopy investigations

**Fig. 5:**
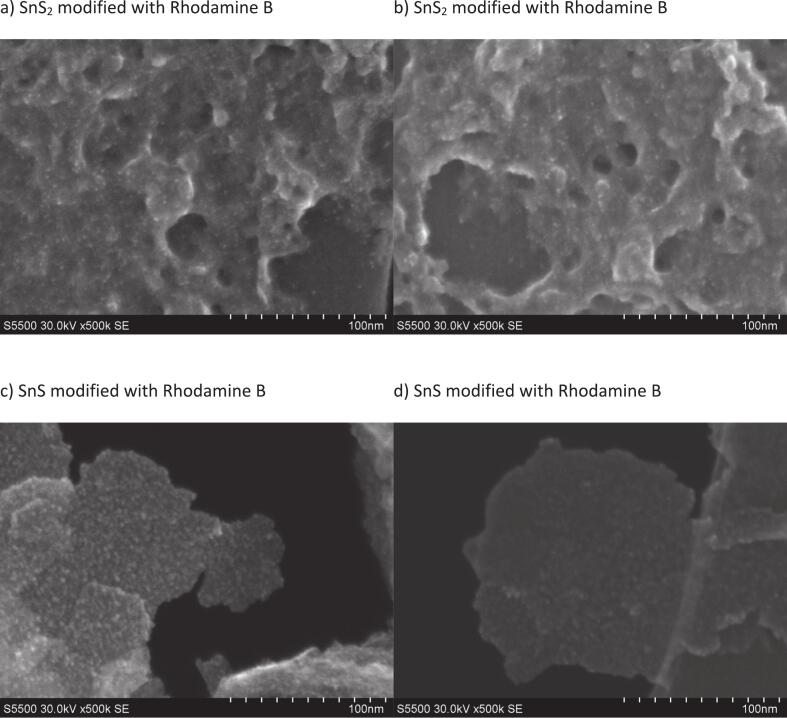
SEM images of dried SnS and SnS_2_ nanopowders modified with Rhodamine B.

Scanning electron microscopy observations were used to study the morphology of synthesized tin sulphides. The SEM images (Fig. 5) confirms the presence of nanograins (bright, white, and small dots in SEM images) in prepared SnS and SnS_2_ suspensions. Nanograins have few nanometers in size. The EDX elemental analyses confirmed the stoichiometry of SnS and SnS_2_ in relevant products of syntheses. Sn:S atomic ratio for SnS sample was 0.97, while for SnS_2_ sample it was 0.52. Both values are near the ideal stoichiometry.

### 3.5 Transmission electron microscopy investigations

**Fig. 6:**
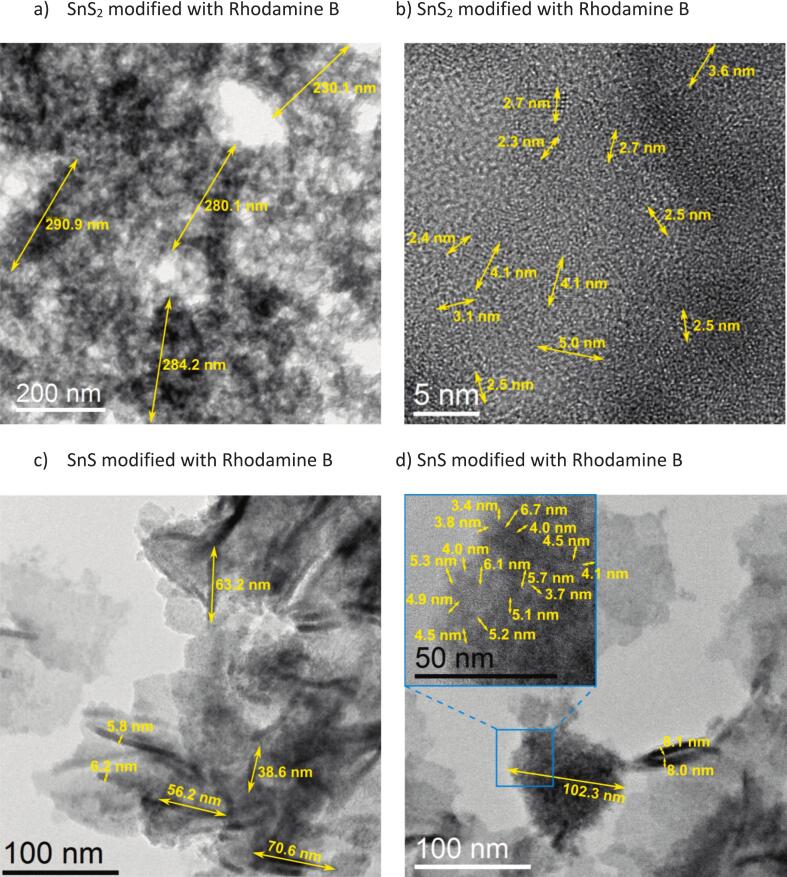
TEM images of SnS and SnS_2_ samples.

**Fig. 7:**
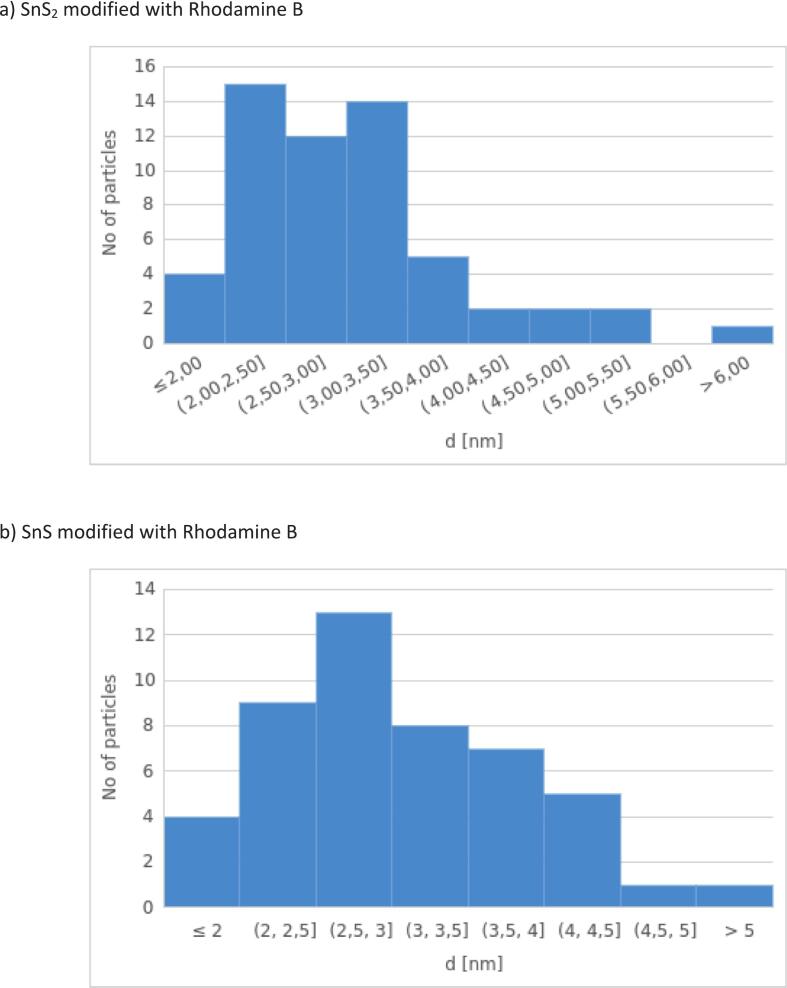
Histograms of nanocrystallites sizes for: a) SnS_2_ modified with Rhodamine B, b) SnS modified with Rhodamine B, based on overview TEM images.

Morphology of the powders was additionally evaluated with the use of TEM investigation. Overview images of the samples, collected at lower magnifications, confirm the presence of the nanograins for both samples (Fig. 6). Nanograins are tightly packed within extensive agglomerates that reach even 400 nm in size for the largest dimension. In the case of SnS sample, aside from the nanocrystalline agglomerates, nanowire-like forms were also observed. The length of the nanowires typically do not exceed 100 nm, with individual ones reaching even 200 nm, while the diameter is around 6–7 nm. The local elemental analysis with the use of EDX spectroscopy confirmed the stoichiometry of SnS_2_ – the mean Sn:S ratio was 0.55. For SnS nanopowder, its stoichiometry was confirmed both in the case of agglomerated nanograins and nanowires, and the mean Sn:S ratio was 1.22. The difference from ideal stoichiometry can result from extremely small dimensions of the nanograins and disturbance from surrounding material. The histograms of sizes of nanocrystallites in modified samples are presented in Fig. 7, showing that in the case of modified SnS_2_ sample the size was typically in range 2.0–3.5 nm while for the modifed SnS sample the size was mostly in range 2.0–4.5 nm.

**Fig. 8:**
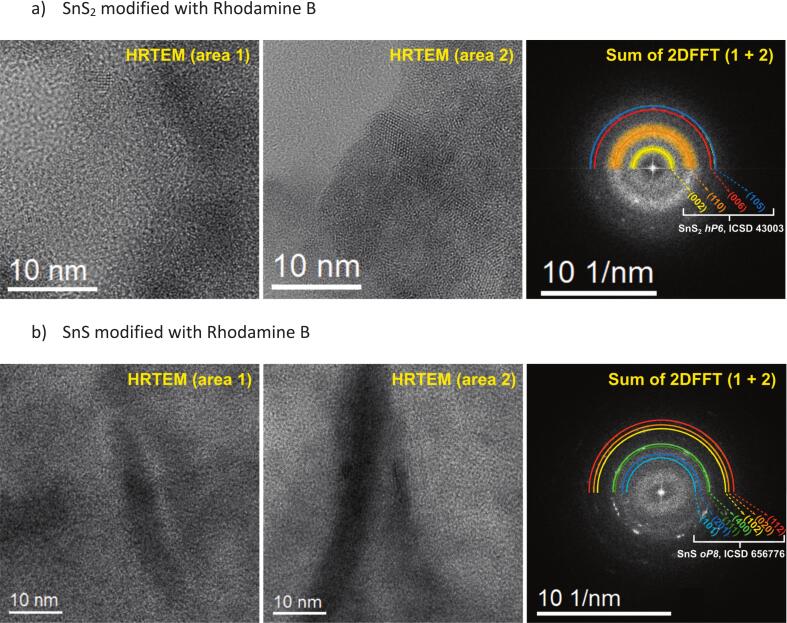
HRTEM images of the SnS_2_ and SnS samples and the sums of their 2DFFT with interpretation.

In order to establish the crystal structure of the SnS and SnS_2_ in the investigated nanopowders, samples of them were subjected to the HRTEM observations. Firstly, the analysis was performed based on the 2D Fast Fourier Transforms of the high-resolution images of polycrystalline areas of the samples (Fig. 8). In the case of SnS_2_ sample the 2DFFT image shows rings corresponding to characteristic reflexes of the SnS_2_ hP6 structure. It is worth noting that the rings corresponding to (002) and (110) reflexes are broad and diffuse, indicating nanoamorphous character of the investigated powder, similarly to the XRD data. For the SnS sample, observed rings correspond to the reflexes of SnS oP8 structure. They are visibly sharper and more numerous which suggest higher crystallinity of this material.

**Fig. 9:**
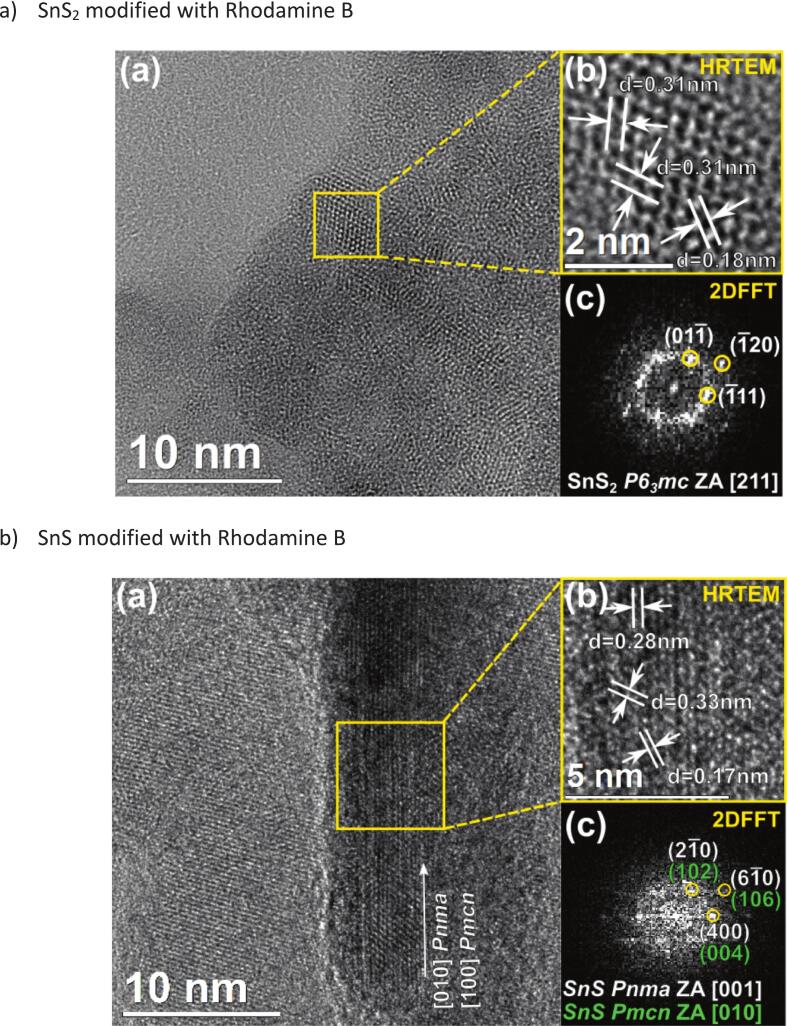
Structural analyses of SnS and SnS_2_ nanocrystals modified with Rhodamine B, based on HR-TEM images.

Further investigation of the HRTEM images involved analysis of the structure of individual nanocrystals (Fig. 9). In the case of SnS_2_ nanocrystal, the interplanar distances and angles measured from the HRTEM image are in agreement with the literature values of these parameters for hexagonal SnS_2_ crystal structure (hP6) with space group P6_3_mc and unit cell parameters a = 3.645 Å, c = 11.802 Å (ICSD 43003). For HRTEM image of SnS nanocrystal the analysis was performed in the same way. Measured values of the distances and angles between planes correspond to the literature values of the orthorhombic SnS structure (oP8) of two possible space groups – Pnma with unit cell parameters a = 11.201 Å, b = 3.990 Å, c = 4.335 Å (ICSD 656776) and Pmcn with unit cell parameters a = 3.990 Å, b = 4.340 Å and c = 11.200 Å (ICSD 30271).

### 3.6 X-ray photoelectron spectroscopy investigations

The adsorption of the Rhodamine B (RhB) dye on SnS_2_ and SnS quantum dots was confirmed by XPS spectral analysis. Table 1 presents the elemental composition (at %) of the studied samples. From these values, the relative amount of RhB anchored to the quantum dots was calculated, revealing a higher adsorption capacity for SnS_2_ (approximately 6 % RhB) compared to SnS (around 4 % RhB).

**Table 1:** Results of the XPS elemental composition (at %) of tested samples from high resolution spectra.

Sample	C 1 s [%]	N 1 s [%]	O 1 s [%]	Cl 2p [%]	S 2p [%]	Sn 3d [%]
Rhodamine B **(RhB)**	82.3	5.0	11.0	1.7	–	–
**SnS_2_ modified**	32.7	0.4	38.0	3.2	12.5	13.4
**SnS modified**	28.6	<0.4	40.0	2.3	7.4	21.8
**SnS modified (after 5 cycles)**	59.6	0.5	23.9	Below detection limit	3.4	12.6

**Fig. 10:**
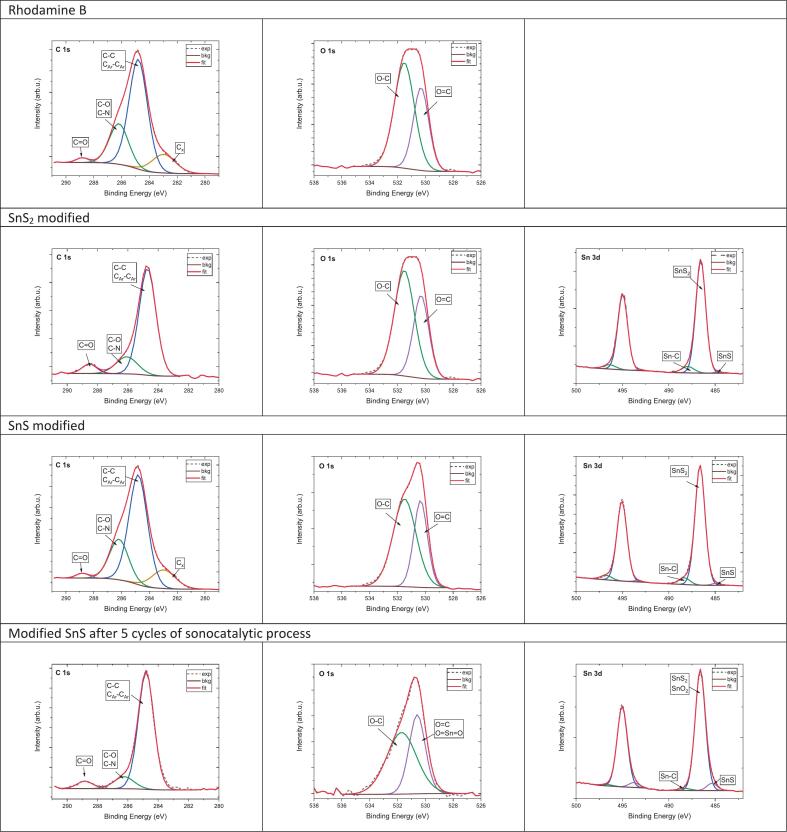
XPS spectra of Rhodamine B and analysed modified SnS_2_ and SnS samples (including sample after 5 cycles of sonocatalytic process). For the Rhodamine B the C_x_ and O_x_ components in C 1 s and O 1 s lines, respectively, come from the charging effect.

Examination of the C 1 s and O 1 s spectra (Fig. 10, Table 2, Table 3) revealed difference between free and bound RhB molecules. Before being linked to the tin sulphide quantum dots, the free dye molecules exhibited strong hydrogen bonding. This hydrogen bonding influenced the electron density around the C=O bond, weakening it and leading to bond elongation. Upon attaching the RhB molecules to the tin sulphide nanodots, this effect partially disappeared. This de-shielding effect is evidenced by an increase in the peak area associated with the C=O (carbonyl) moiety.

**Table 2:** XPS data analysis of C 1 s line. The binding energy (eV); percentage of given fraction (at %) and full width at half maximum (FWHM, eV) for components in analyzed samples.

Sample	C-C		C-O, C-N		C=O	
	Energy FWHM	at %	Energy FWHM	at %	Energy FWHM	at %
Rhodamine B (**RhB)**	284.8 1.6	71.0	286.2 1.6	27.0	288.8 1.0	2.0
**SnS_2_ modified**	284.8 1.3	78.0	286.1 1.7	16.0	288.6 1.1	6.0
**SnS modified**	284.8 1.2	66.9	286.0 1.7	22.6	288.7 1.4	10.5
**SnS modified (after 5 cycles)**	284.8 1.2	83.8	286.2 1.5	10.9	288.8 1.2	5.3

**Table 3:** XPS data analysis of O 1 s line. The binding energy (eV); percentage of given fraction (at %) and full width at half maximum (FWHM, eV) for components in analyzed samples.

Sample	O=C		O-C	
	Energy FWHM	at %	Energy FWHM	at %
Rhodamine B (**RhB)**	531.8 1.5	33.3	533.2 2.1	66.7
**SnS_2_ modified**	530.3 1.3	37.7	531.5 1.7	62.3
**SnS modified**	530.4 1.1	36.9	531.5 2.0	63.1
	O=C/O=Sn=O		O-C	
**SnS modified (after 5 cycles)**	530.6 1.4	40.8	531.5 2.5	59.2

To understand the interaction between the molecules of RhB dye and the tin sulphide nanodots, the Sn 3d spectra of the dye-modified SnS and SnS_2_ nanodots (Fig. 10) were compared with those of the unmodified nanodots previously reported [37].

This comparison showed that the RhB dye molecule binds to the Sn atom, as evidenced by the Sn-C interaction, observed for both samples (Fig. 10). The analysis revealed the presence of an Sn-C component at around 488 eV [63], at the percentage level equal to 6.6 % and 5.9 % for modified SnS_2_ and SnS samples, respectively. Moreover, after the adsorption of the RhB dye molecule, the tin content in the + 2 oxidation state decreased to around 1 % for both analysed samples.

### 3.7 Determination of energy bandgap value using Tauc method

Registered UV–Vis spectra of purified suspensions resulting from sonochemical syntheses were used to perform the Tauc analyses and determine the optical energy band gaps. The Tauc analyses were narrowed to only two allowed transitions, direct and indirect, as forbidden transitions are not chemically meaningful in the context of catalytic experiments. The raw UV–Vis spectra of modified SnS and SnS_2_ quantum dots are presented in Fig. S1. They indicate the existence of additional absorption peak at c.a. 570 nm resulting from the modification of nanopowders with RhB. This peak is not observed for SnS samples due to the large absorption of light by SnS quantum dots in this region.

**Fig. 11:**
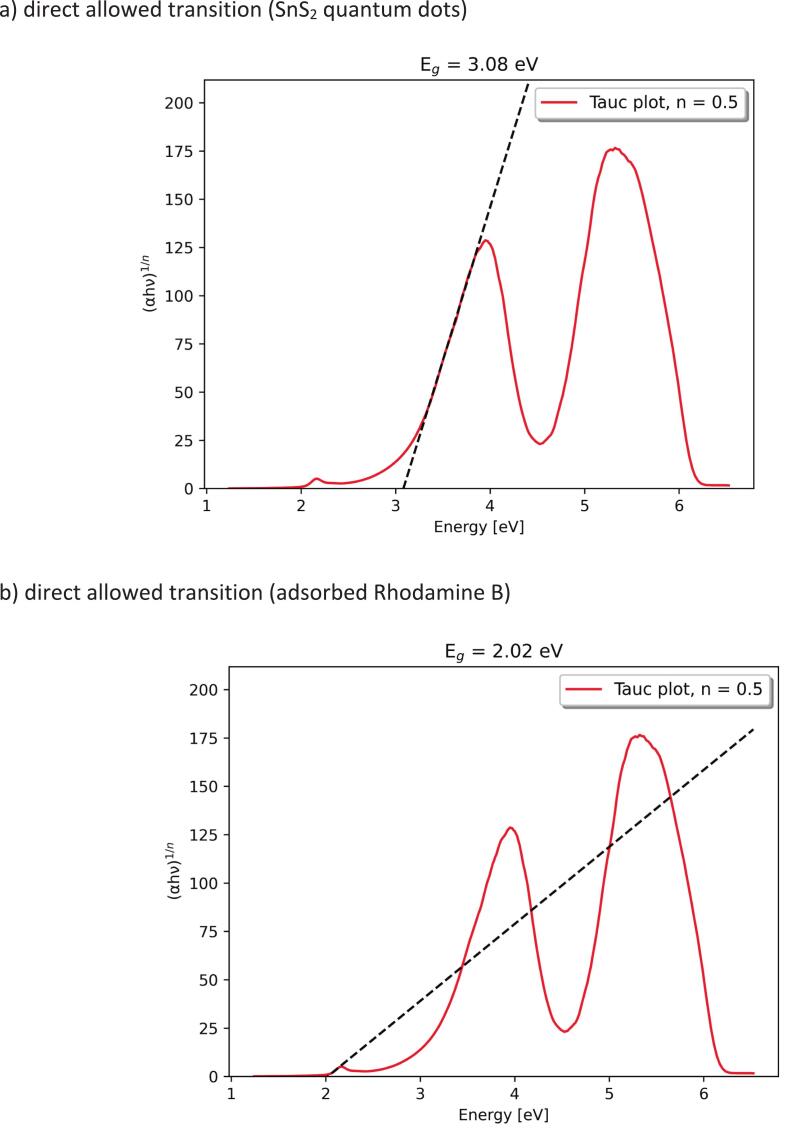

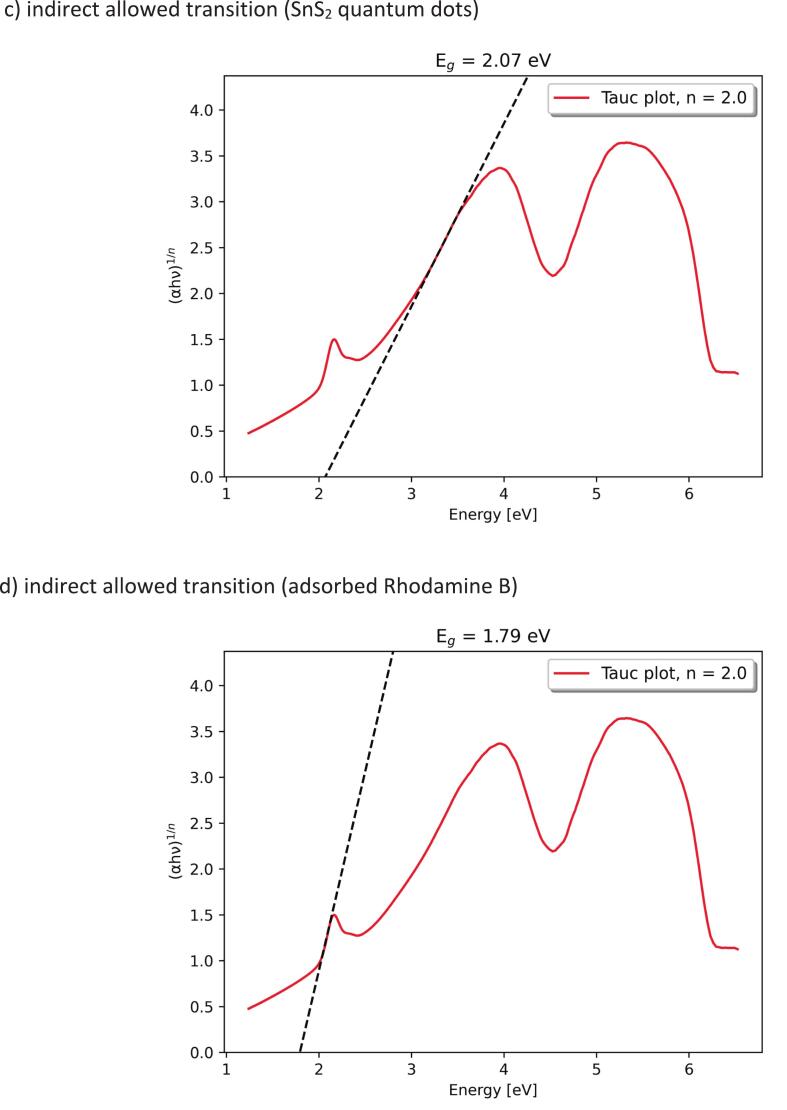
Tauc plots for direct and indirect allowed optical transitions for sonochemically obtained SnS_2_ quantum dots modified with Rhodamine B using amount of 5 mg of RhB.

The results of analyses based on the Tauc method show that the modification with Rhodamine B resulted in the presence of additional electronic transition in samples, corresponding to the Rhodamine B molecules. Fig. 11 presents a comparison of the Tauc plots for indirect allowed and direct allowed transitions for SnS_2_ modified while taking 5 mg of Rhodamine B into synthesis. Plots for other samples are very similar so they are omitted for clarity.

The determined values of optical energy bandgaps for each synthesized sample are collected in Table 4. The values for direct allowed transition for SnS_2_ samples lie in range 3.08 – 3.37 eV indicating significant quantum confinement effect as the bandgap of bulk crystalline SnS_2_ is c.a. 2.4 eV [64], [65], [66]. Interestingly, the samples modified with larger amount of Rhodamine B (greater than 5 mg, that is for 10, 15, and 20 mg) exhibited increased value of optical energy bandgap for this transition (from 3.08 eV to 3.29 – 3.37 eV). The values of indirect allowed transition for SnS_2_ samples lie in range 1.8 – 2.09 eV, however this values can’t be compared to previous studies due to lack of corresponding literature. The values for direct allowed transition for SnS samples lie in range 1.93 – 2.87 eV also indicating the quantum confinement effect as the bandgap for bulk crystalline SnS is c.a. 1.3 eV [67], [68], [69], [70]. The indirect transition for SnS samples are in range from 0.85 to 1.26 eV; such values are quite similar to the indirect bandgap of bulk SnS which is 1.09 eV, however they still indicate quantum confinement effect for sample with indirect bandgap of 1.26 eV [69], [70], [71].

**Table 4:** Collection of values of optical energy bandgaps for all synthesized SnS and SnS_2_ samples.

Sample	Direct allowed transition	Indirect allowed transition
SnS_2_ modified with 5 mg of Rhodamine B	3.08 eV (SnS_2_) 2.02 eV (RhB)	2.07 eV (SnS_2_) 1.79 eV (RhB)
SnS_2_ modified with 10 mg of Rhodamine B	3.37 eV (SnS_2_) 2.04 eV (RhB)	2.09 eV (SnS_2_) 1.82 eV (RhB)
SnS_2_ modified with 15 mg of Rhodamine B	3.33 eV (SnS_2_) 2.04 eV (RhB)	1.97 eV (SnS_2_) 1.84 eV (RhB)
SnS_2_ modified with 20 mg of Rhodamine B	3.29 eV (SnS_2_) 2.03 eV (RhB)	1.8 eV (SnS_2_) 1.83 eV (RhB)
SnS modified with 5 mg of Rhodamine B	2.16 eV (SnS)	1.26 eV (SnS)
SnS modified with 10 mg of Rhodamine B	2.87 eV (SnS) 1.92 eV (RhB)	0.85 eV (SnS)
SnS modified with 15 mg of Rhodamine B	2.8 eV (SnS) 2.09 eV (RhB)	1.03 eV (SnS) 1.45 eV (RhB)
SnS modified with 20 mg of Rhodamine B	1.93 eV (SnS) 1.95 eV (RhB)	1.0 eV (SnS) 1.28 eV (RhB)

Among samples additional transitions occur resulting from the modification of quantum dots with Rhodamine B. Direct allowed transition corresponding to this modification has quite similar bandgap among samples and varies slightly in range 1.92 – 2.09 eV. Relevant indirect allowed transition has bandgap in range from 1.79 to 1.84 eV for modified SnS_2_ samples and from 1.28 to 1.45 eV for modified SnS samples. Thus the lowering of bandgap for this transition is accented for modified tin(II) sulphide quantum dots.

### 3.8 Mott-Schottky analyses

**Fig. 12:**
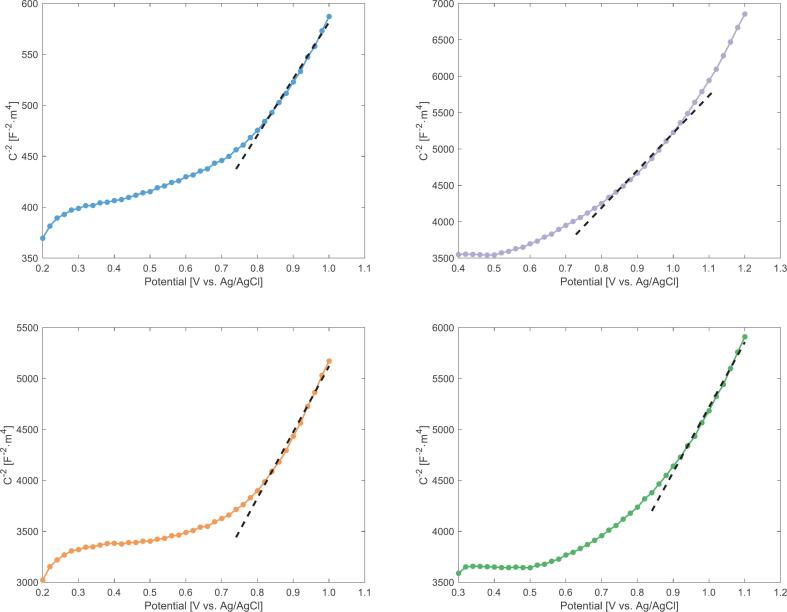
Mott-Schottky plots of the SnS and SnS_2_ samples performed in dark at a frequency of 1 kHz with an AC amplitude of 10 mV, in a 0.1 M KCl electrolyte: unmodified SnS (top left corner), modified SnS_2_ (top right corner), modified SnS (bottom left corner), unmodified SnS_2_ (bottom right corner).

The Mott-Schottky plots of the samples examined are presented in Fig. 12. A positive slope indicates the n-type nature of every material tested. To estimate samples’ E_FB_, linear regions of the obtained curves were identified and approximated with linear functions, with R^2^ coefficients over 0.99 for each sample, and then extrapolated to the potential axis of the Mott-Schottky plot. The analyses revealed E_FB_ values of −0.048, −0.017, 0.208, and 0.182 V vs. Ag/AgCl electrode for the unmodified SnS, modified SnS_2_, modified SnS, and unmodified SnS_2_ samples, respectively.

Having the flat band potentials for catalysts, it is possible to calculate the energies of conduction (E_CB_) and valence (E_VB_) bands, according to the following equations [72]:

(8) $$E_{\mathrm{CB}}=E_{\mathrm{FB}}+\Delta E-0.059\left(7-pH\right)$$

(9) $$E_{\mathrm{VB}}=E_{\mathrm{CB}}+E_{g}e$$

Where:

pH- is the pH of the solution

$E_{g}$- is the bandgap of material.

ΔE- is the standard potential of Ag/AgCl reference electrode for the normal hydrogen electrode (NHE) (value taken as 0.21 V).

The calculated E_CB_ and E_VB_ values for unmodified and modified catalysts are collected in Table 5.

**Table 5:** Collection of values of flat-band potentials and energies of conduction and valence bands for both SnS and SnS_2_ modified and unmodified.

Catalyst	E_FB_ [V] vs Ag/AgCl	E_g_ [V]	E_CB_ [V] vs NHE	E_VB_ [V] vs NHE
SnS	−0.048	1.05	0.089	1.139
SnS_2_	0.182	2.70	0.319	3.019
SnS modified	0.208	1.93	0.345	2.275
SnS_2_ modified	−0.017	3.29	0.120	3.410

### 3.9 Contact angle measurements

The results of contact angle measurements are presented in Table 6. In the case of modified SnS_2_ quantum dots there is no clear trend regarding degree of modification and contact angle, however for modified samples contact angles were greater than in the case of unmodified SnS_2_. This indicates the decrease in wettability after functionalization with Rhodamine B. On the other hand, in the case of SnS quantum dots, the modified samples exhibited extraordinary wettability which made the contact angle measurements impossible – no drop was formed for each applied solvent. Only in the case of unmodified SnS sample the measurements were possible but only for water and glicerine, not for diiodomethane. The unmodified SnS showed better wettability than each of SnS_2_ samples (regardless modification).

**Table 6:** Results of the contact angle measurements.

Solvent / contact angle [°]	Sample					
	SnS_2_ unmodified	SnS_2_ 5 mg RhB	SnS_2_ 10 mg RhB	SnS_2_ 15 mg RhB	SnS_2_ 20 mg RhB	SnS unmodified
Water	36	57	54	46	58	31
CH_2_I_2_	35	45	42	37	45	–
Glicerine	50	60	46	51	63	23

### 3.10 Sonocatalytic and photocatalytic activity

**Fig. 13:**
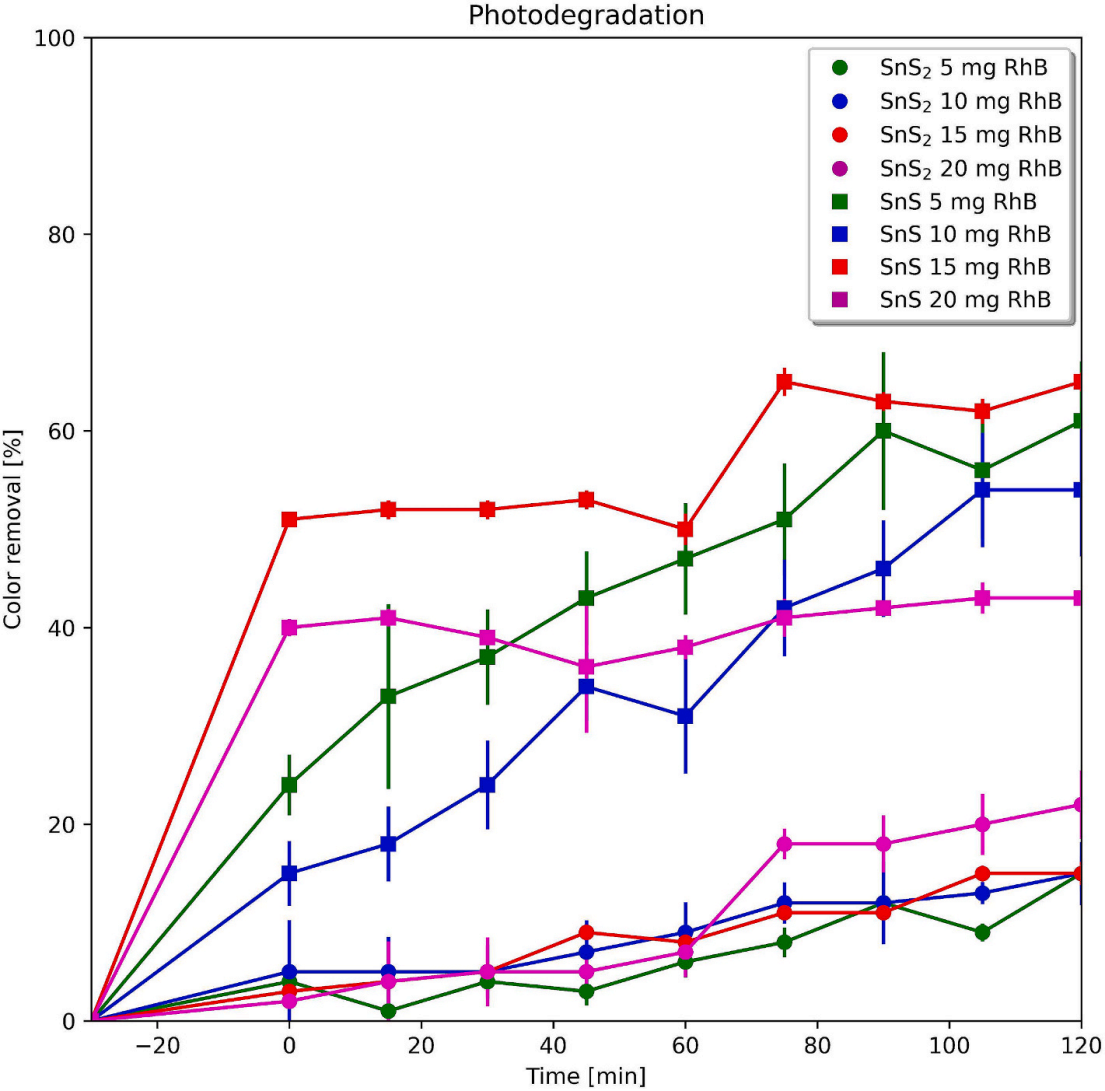
Change of color removal during the photocatalytic process.

Each obtained nanopowder was applied as catalyst in the photo- and sonodegradation of azo-dye Metanil Yellow. Fig. 13 presents plots of color removal during the photocatalytic process for various modified SnS and SnS_2_ quantum dots used as catalysts. The modification with Rhodamine B affected not only the photocatalytic activity, as expected, but also the adsorption. In the case of SnS quantum dots upon slight modification (addition of 5 mg of Rhodamine B to the reaction mixture) the photocatalytic activity increased in comparison to the unmodified SnS. However, with increased degree of modification the photocatalytic activity lowered, but the adsorption increased. In the case of modification of SnS_2_ quantum dots the modification decreased the photocatalytic activity in comparison with unmodified SnS_2_. However, with increased modification the activity was slightly improved (among modified samples).

**Fig. 14:**
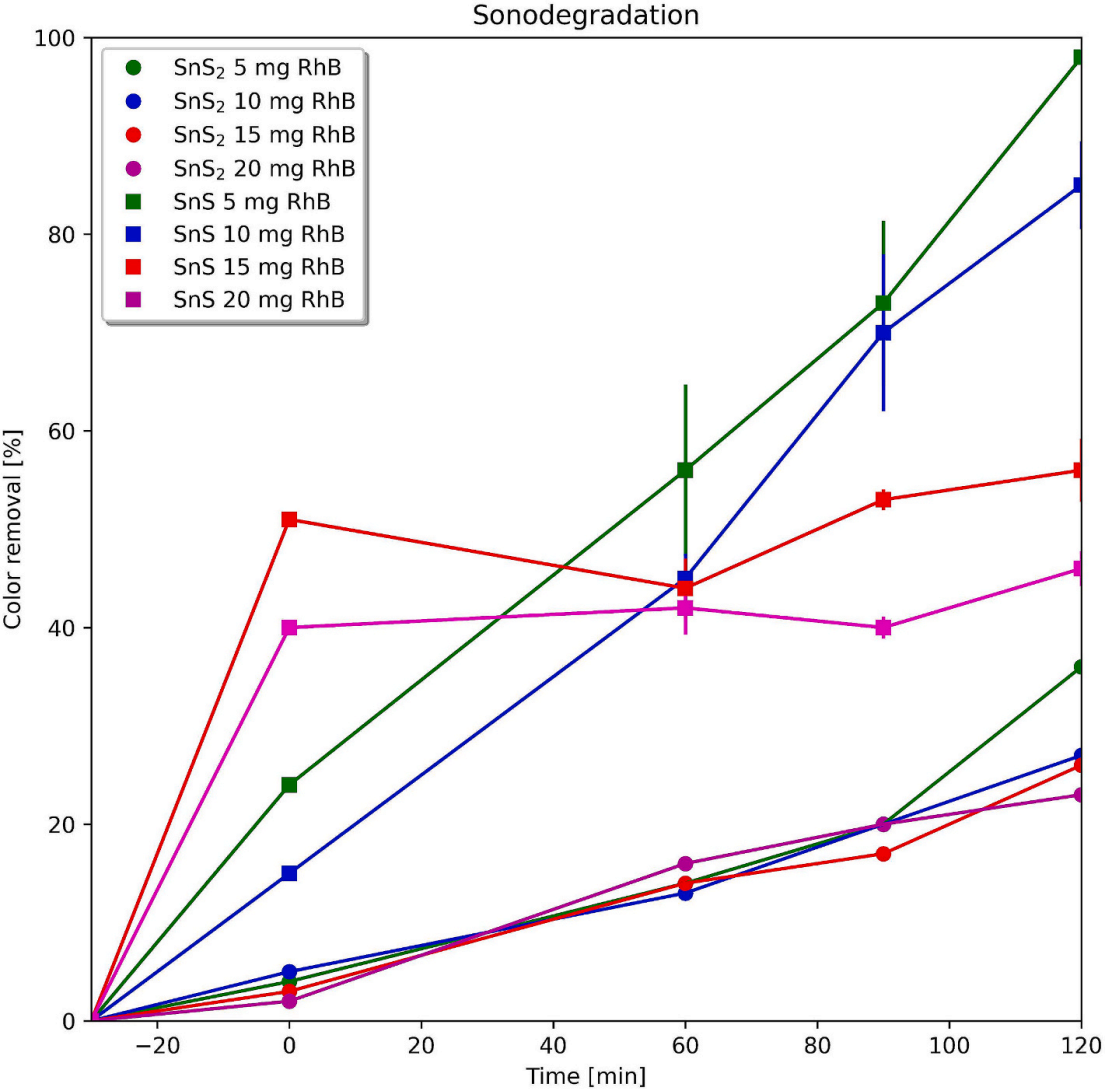
Change of color removal during the photocatalytic process.

Fig. 14 presents similar change of color removal but during the sonocatalytic process. In this process the trends are similar to those observed in the photocatalytic process – the modification with Rhodamine B improved the catalytic activity upon addition of 5 mg of Rhodamine B to the reaction mixture. Using this sonocatalyst almost 100 % decolorization was achieved, after 120 min of sonication. Further increase of degree of modification resulted in decreased activity. Modified SnS_2_ quantum dots showed lowered activity when compared with unmodified SnS_2_. Differences in sonocatalytic activity between modified SnS_2_ samples are negligible.

Table 7 collects data on the overall color removal for each used catalyst and for each catalytic process after 2 h counted from the moment of establishment of the adsorption–desorption equillibrium. The values clearly show that the color removal was increased when using SnS quantum dots modified with 5 mg of Rhodamine B, for both processes. In the case of SnS_2_ quantum dots their catalytic activities lowered which may be caused by blocking of active centers with RhB molecules. This lowering was less pronounced in the sonocatalytic process what suggests the importance of energy bandgap. In the sonocatalytic process the energy bandgap is less important than in the photocatalytic one. However, the modification with Rhodamine B added new transition present in the catalyst but with lower energy than quantum dot exhibits itself. In consequence, the photoexcited holes and electrons may have less energy than they would have in the case of standalone SnS_2_ quantum dot what affects the reactivity. Also, the modification with Rhodamine B lowered the wettability of SnS_2_ quantum dots what is most probably responsible for decreased sonocatalytic activity. In the case of SnS samples the modification with RhB improved the wettability what may explain the improved sonocatalytic activity for SnS modified with 5 mg of RhB. Upon further modification the decrease in sonocatalytic activity may be due to the blockade of active centers. The improved photocatalytic activity may be caused by increased light absorption due to the presence of RhB molecules on the surface of SnS quantum dots. In this case the additional transition resulting from the presence of RhB molecules has quite similar energy compared to bare SnS. Lowering of photocatalytic activity for SnS samples upon further modification may be also due to the blockade of active centers.

**Table 7:** Results of the overall color removals.

Catalyst	Overall color removal (photocatalytic process) [%]	Overall color removal (sonocatalytic process) [%]	Color removal after 30 min of adsorption [%]
SnS unmodified [37]	51	85	5
SnS (5 mg of RhB)	62	98	24
SnS (10 mg of RhB)	54	85	15
SnS (15 mg of RhB)	66	56	51
SnS (20 mg of RhB)	43	46	40
SnS_2_ unmodified [37]	67	38	3
SnS_2_ (5 mg of RhB)	15	36	4
SnS_2_ (10 mg of RhB)	15	27	5
SnS_2_ (15 mg of RhB)	15	26	3
SnS_2_ (20 mg of RhB)	22	23	2

**Fig. 15:**
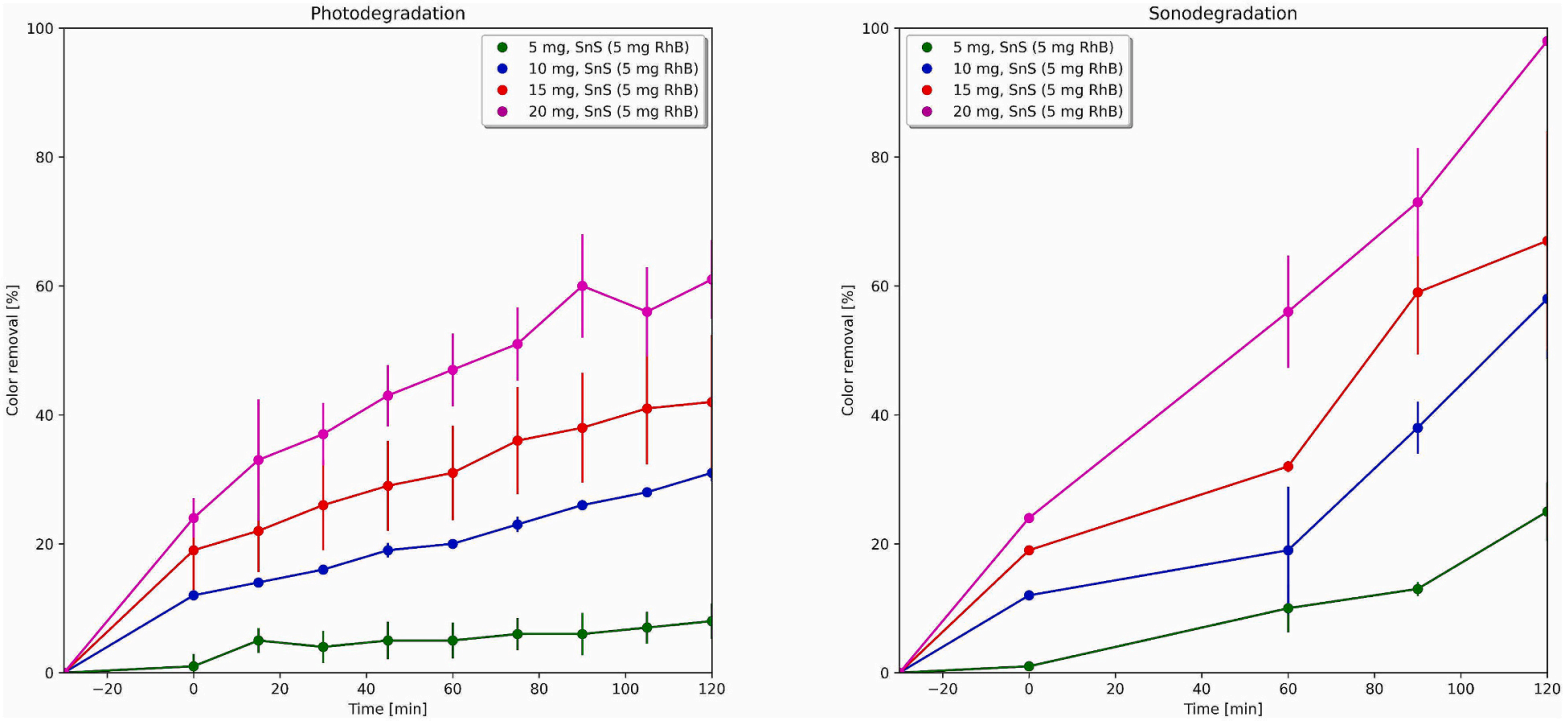
Change of color removal during the photo- and sonocatalytic processes upon varied catalyst amount for SnS quantum dots modified with 5 mg of Rhodamine B.

For the best photo- and sonocatalyst, SnS quantum dots modiified with 5 mg of Rhodamine B, also experiments investigating the influence of amount of catalyst on color removal were performed (Fig. 15). The color removal increased monotonically with the increased amount of catalyst, as expected.

### 3.11 Scavenging experiments

Scavenging experiments were performed to elucidate the degree of involvement of various reactive intermediates – hydroxyl radicals, •OH, superoxide anion radicals, •O_2_^−^, and positive holes, h^+^ − in the sono- and photocatalytic degradations. The experiments were performed for the best catalyst among all investigated (that is, SnS modified with 5 mg of Rhodamine B) and for the unmodified catalysts (SnS, SnS_2_). Scavengers are chemical individuals that react much faster with reactive intermediates than the degraded compound. Upon the addition of scavenger to the reaction mixture the degree of decolorization decreases to the extent determined by the degree of involvement of certain reactive intermediate. The results of scavenging experiments are collected in Table 8, Table 9, Table 10.

**Table 8:** Results of the scavenging experiments for the best catalyst (SnS modified with 5 mg of Rhodamine B).

Scavenger	Reactive intermediate	Decolorization (photocatalytic process) [%]	Decolorization (sonocatalytic process) [%]	Decolorization (from adsorption) [%]
None	−	62	98	24
t-BuOH	•OH	53	89	9
Ascorbic acid	•O_2_^−^	54	61	25
KI	h^+^	56	89	16

**Table 9:** Results of the scavenging experiments for the unmodified SnS catalyst.

Scavenger	Reactive intermediate	Decolorization (photocatalytic process) [%]	Decolorization (sonocatalytic process) [%]
None	–	51	85
t-BuOH	•OH	32	33
Ascorbic acid	•O_2_^−^	11	15
KI	h^+^	34	72

**Table 10:** Results of the scavenging experiments for the unmodified SnS_2_ catalyst.

Scavenger	Reactive intermediate	Decolorization (photocatalytic process) [%]	Decolorization (sonocatalytic process) [%]
None	–	67	38
t-BuOH	•OH	28	14
Ascorbic acid	•O_2_^−^	10	13
KI	h^+^	26	13

Results presented in Table 8, Table 9, Table 10 indicate that the most important reactive intermediate is the superoxide anion radical for both processes, for each catalyst. However this result isn’t in agreement with the calculated energies of valence and conduction bands for unmodified SnS and SnS_2_, and modified SnS (Table 5). The standard potentials for O_2_/O_2_^•-^ and H_2_O/^•^OH pairs are c.a. −0.33 V and 2.31 V (vs normal hydrogen electrode), respectively [72], [73]. None of these three catalysts have sufficient value of conduction band energy to make the production of superoxide anion radical possible. On the other hand, the production of hydroxyl radicals by each of these catalysts is still possible. The potential for H_2_O/^•^OH pair is pH sensitive and under the conditions present in this study (pH c.a. 5.76) it’s potential is slightly below 2 V, according to the Nernst equation:

(10) $$E=E^{0}-\frac{0.059}{n}\cdot m\cdot pH$$

Where:

E- potential [V].

$E^{0}$- standard potential [V].

n- number of electrons included in electrochemical reaction

m- number of protons included in electrochemical reaction

**Fig. 16:**
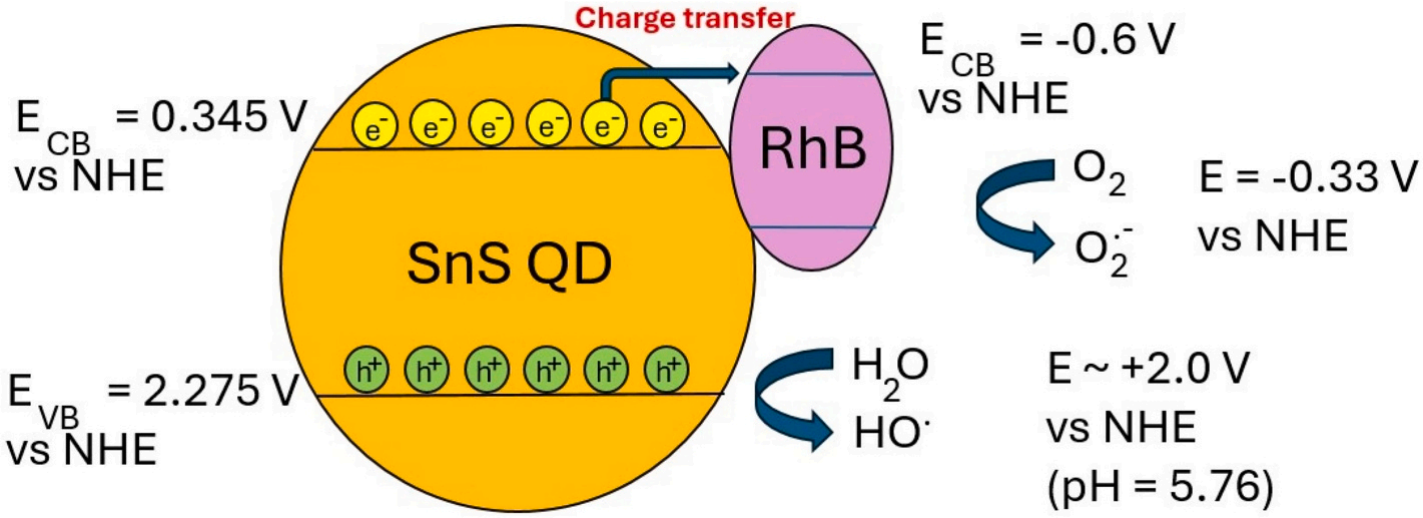
Schematic diagram of charge transfer in excited SnS quantum dot modified with Rhodamine B.

The possible explanation for the importance of superoxide anion radical during the processes is the secondary production of it (e.g. by hydroxyl radicals), instead of direct production by excited photo- or sonocatalyst. For most of catalysts, the energy of valence band lies above 2 V, so the positive holes have enough energy to oxidize water molecules and produce hydroxyl radicals. This is not the case for unmodified SnS, however it still shows significant photo- and sonocatalytic degradation. It is possible that also other effects, like wettability (especially in the case of sonoprocess), indirect bandgap or impurities (like marginal amounts of tin oxides), may contribute to the processes. Also, for the quantum dots modified with Rhodamine B, it is proved that electron transfer occurs from semiconducting quantum dot to the Rhodamine B molecule [51], [52]. The energy of conduction band is c.a. −0.6 V vs NHE so electrons present in this band may take part in the reduction of oxygen to superoxide anion radical (Fig. 16) [74], [75]. However, this explanation still cannot be applied for the case of unmodified SnS and SnS_2_ quantum dots.

### 3.12 Stability analysis of the best catalyst and it’s characterization after the process

Cycle test was performed for the best catalyst among prepared samples (SnS modified with 5 mg of Rhodamine B). It was used in five subsequent cycles. Results presented in Table 11 indicate the decrease of catalyst’s activity in next cycles upon reaching total deactivation.

**Table 11:** Percentage decolorization in subsequent cycles of sonocatalytic process using SnS quantum dots modified with 5 mg of Rhodamine B.

**Cycle**	1	2	3	4	5
**Decolorization [%]**	98	39	26	26	12

#### 3.12.1 Raman spectroscopy

After the sonocatalytic process, the luminescence for the sample with modified SnS nanograins decreased (Fig. 4 a, b), but it’s intensity remains high. The Raman signal from SnS nanograins is also slightly weaker (Fig. 4 c), which is due to chemical processes occurring on their surface.

#### 3.12.2 X-ray photoelectron spectroscopy

To evaluate the stability and surface changes of the modified SnS quantum dots after the sonochemical degradation of Metanil Yellow, the spent catalyst was re-examined using XPS analysis. As shown in Table 1, the elemental composition of the SnS sample underwent significant changes. A dramatic increase in the carbon content (from 28.6 at % for SnS modified to 59.6 at % for modified SnS after 5 cycles) was observed, accompanied by a simultaneous decrease in the atomic percentages of Sn (from 21.8 % to 12.6 %) and S (from 7.4 % to 3.4 %). This phenomenon indicates the intensive adsorption of Metanil Yellow molecules or their organic degradation intermediates onto the catalyst surface, creating a carbon-rich shielding layer that attenuates the photoelectron signals from the underlying tin sulphide core. This is further supported by the C 1 s line deconvolution (Table 2, Fig. 10), which revealed a notable increase in the C-C fraction from 66.9 % to 83.8 %, typical for accumulated organic species.

Concurrently, the chlorine content in the sample of modified SnS after 5 cycles dropped below the detection limit, suggesting either complete leaching or the ion exchange of Cl^−^ counterions with ions from the aqueous solution during the catalytic process. The presence of newly adsorbed azo dye on the surface was also manifested by a slight increase in nitrogen (N 1 s) content to a level of 0.5 at %.

Regarding the O 1 s and Sn 3d spectra (Fig. 10, Table 3), a definitive differentiation between the organic species and potential oxide phases (such as SnO_2_) is physically constrained due to severe peak overlapping. In the O 1 s region, the binding energy of the organic O=C bonds coincides with the oxide O=Sn=O component. Similarly, in the Sn 3d spectrum, the signals for SnS_2_ and SnO_2_ exhibit an extreme proximity, with a chemical shift of merely ∼ 0.1 eV [76], [77]. Furthermore, the surface sensitivity of XPS means that the thick carbonaceous layer from the accumulated dye significantly attenuated the photoemission signals from the underlying catalyst core. Therefore, relying on the unequivocal structural evidence from complementary XRD and TEM analyses, these high-resolution components were assigned as combined contributions, designated as O=C/O=Sn=O (for O 1 s) and SnS_2_/SnO_2_ (for Sn 3d). Following this combined assignment, the total surface oxygen content decreased from 40.0 % to 23.9 %, reflecting the extensive coverage of the catalyst surface by the organic degradation products.

#### 3.12.3 Powder X-ray diffraction

**Fig. 17:**
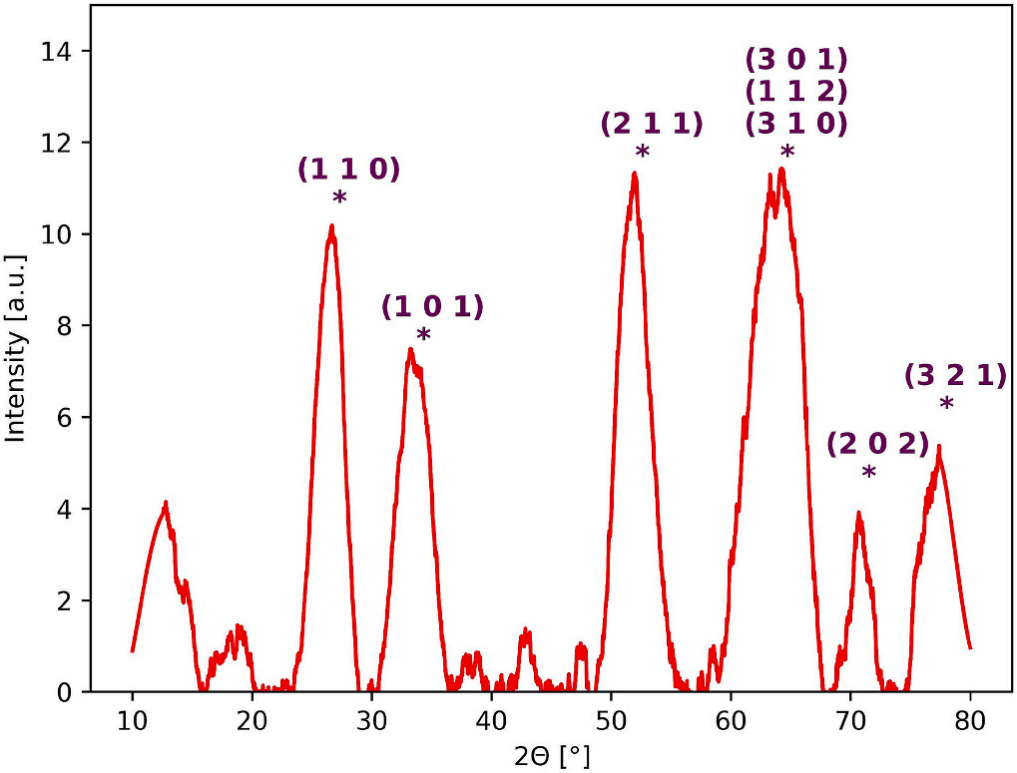
The powder X-ray diffractogram of the best sonocatalyst after 5 cycles.

The powder X-ray diffraction investigation of catalyst after the sonocatalytic processes reveals the presence of nanocrystalline SnO_2_ in the sample (Fig. 17). The observed reflexes math very well with those presented previously in literature [78], [79]. There are still some unrecognized reflexes, but they are relatively small and marginal.

#### 3.12.4 Scanning electron miscroscopy and energy-dispersive X-ray spectroscopy

**Fig. 18:**
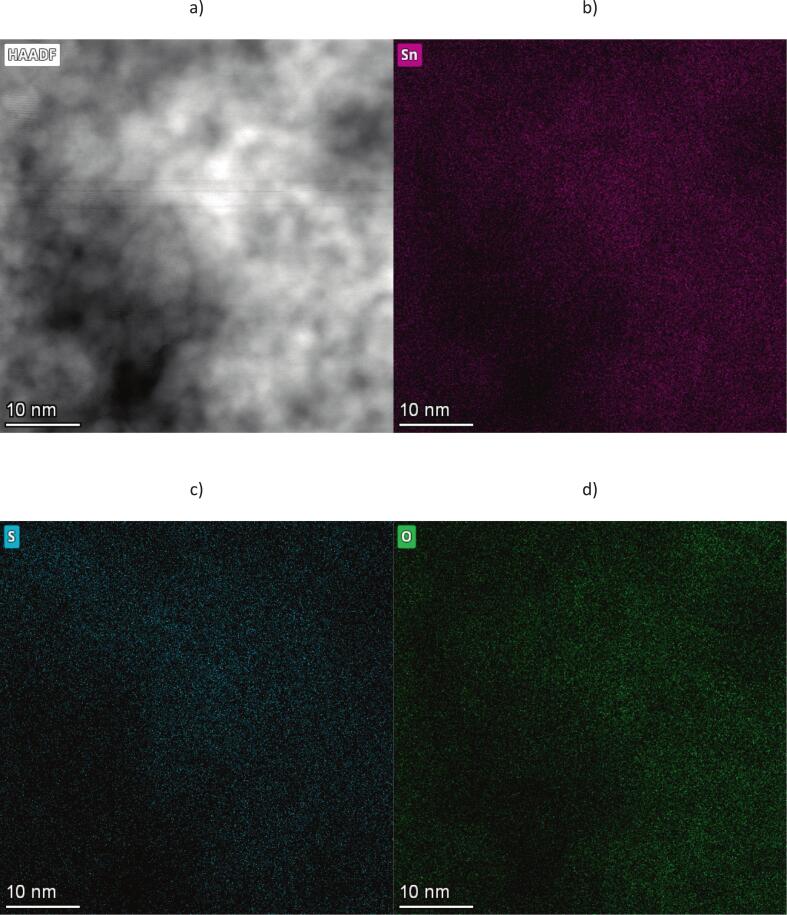
The SEM image (a) and elemental mapping (b-d) of modified SnS after 5 cycles of sonocatalytic process.

The SEM observations of catalyst after 5 cycles shows that overall morphology of dried powder didn’t change. However the elemental composition of catalyst changed drastically showing much higher oxygen content than before the process (Fig. 18). This result is in line with the result of PXRD study, additionally confirming the presence of SnO_2_.

#### 3.12.5 High resolution transmission electron microscopy

**Fig. 19:**
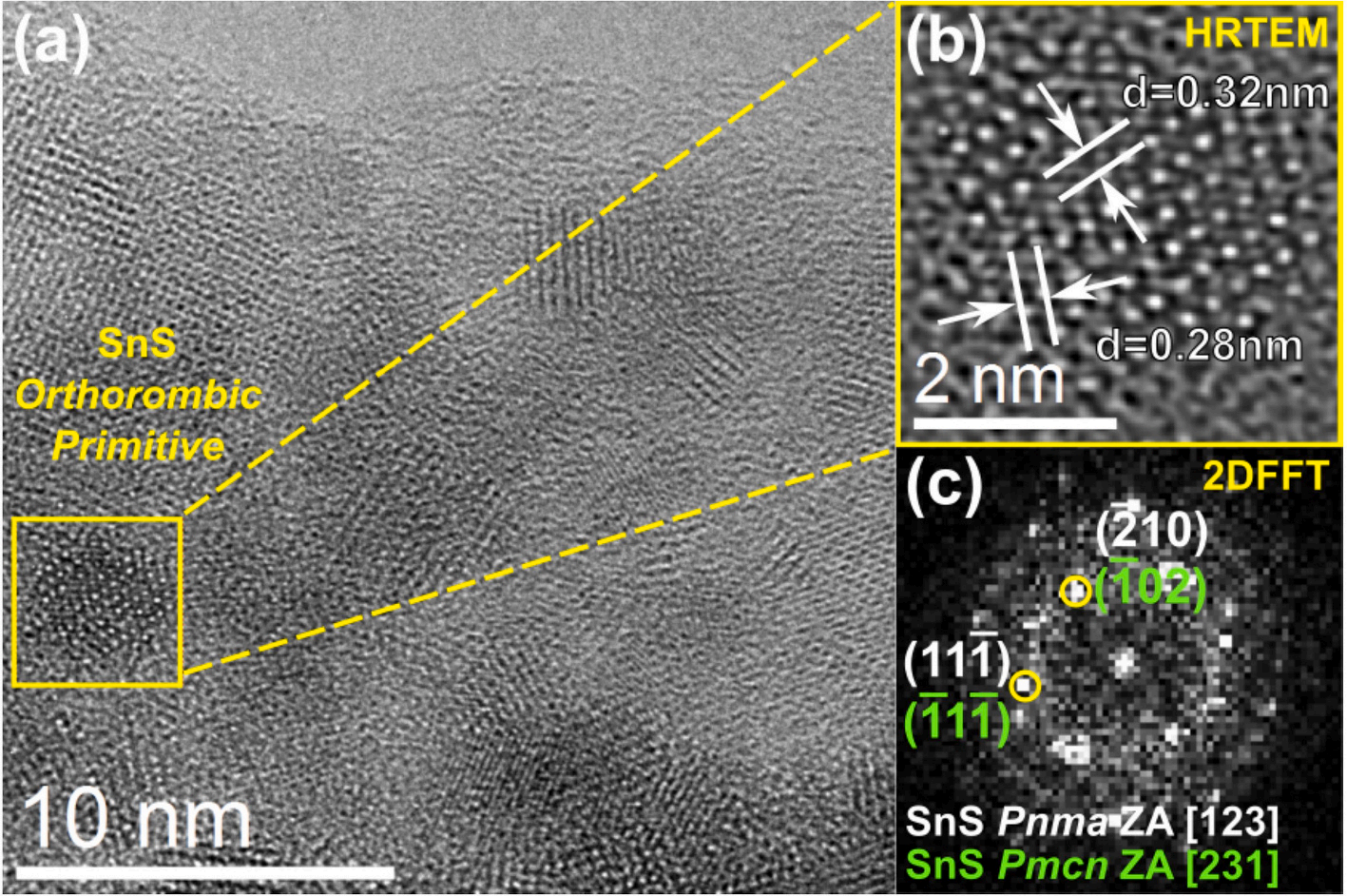
Structural analysis of an SnS nanocrystal in sample after 5 cycles of sonoprocess: (a) HRTEM image with crystal selected for analysis highlighted, (b) magnified image of the selected nanocrystal with measured interplanar distances, (c) 2D-FFT of the image with assigned Miller indices.

The HR-TEM observations revealed the presence of both SnS and SnO_2_ nanocrystals in the catalyst after 5 cycles of sonocatalytic process. Fig. 19 shows structural analysis of a representative SnS nanocrystal observed in the sample. Based on the measured interplanar spacings and angles, the nanocrystal was identified as primitive orthorhombic SnS. The experimental data are consistent with two possible space groups, Pnma (with lattice parameters a = 11.201 Å, b = 3.990 Å, c = 4.335 Å; ICSD Col. Code − 656776) and Pmcn (with lattice parameters a = 3.99 Å, b = 4.34 Å c = 11.200 Å; ICSD Col. Code − 30271), which cannot be distinguished based on the available HRTEM analysis in this case.

**Fig. 20:**
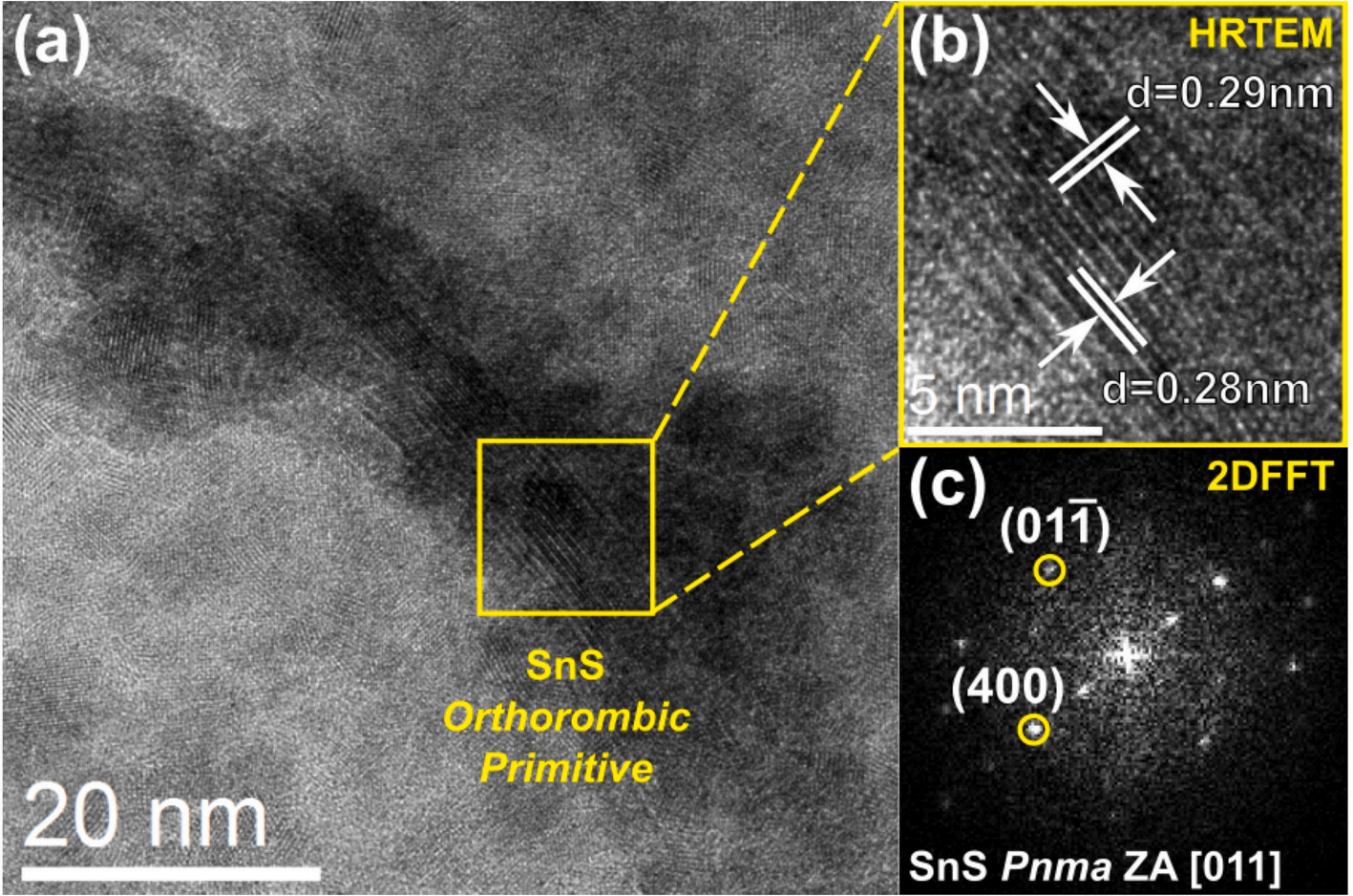
Structural analysis of another SnS nanocrystal in sample after 5 cycles of sonoprocess: (a) HRTEM image with crystal selected for analysis highlighted, (b) magnified image of the selected nanocrystal with measured interplanar distances, (c) 2D-FFT of the image with assigned Miller indices.

Fig. 20 shows the structural analysis of another representative nanocrystal observed in the sample. The measured interplanar spacings and FFT analysis allowed to determine the nanocrystal as orthorhombic primitive SnS. In this case, the experimental data were sufficient to unambiguously distinguish between two candidate space groups, confirming space group Pnma.

**Fig. 21:**
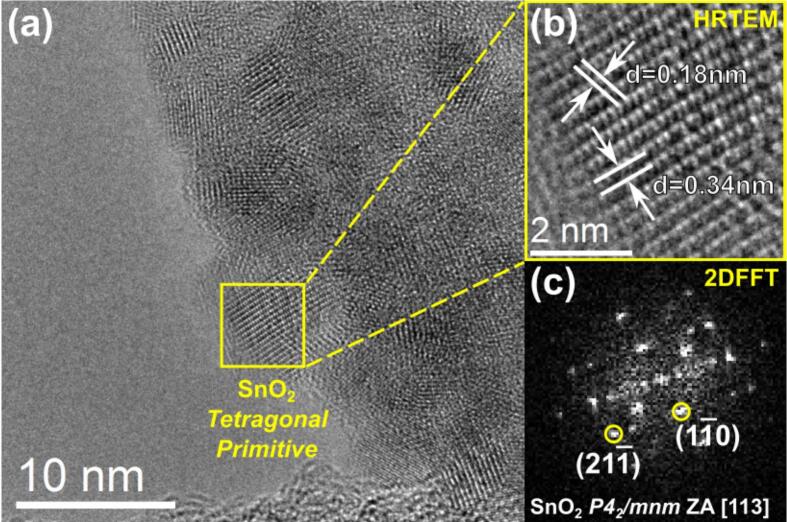
Structural analysis of an SnO_2_ nanocrystal in sample after 5 cycles of sonoprocess: (a) HRTEM image with crystal selected for analysis highlighted, (b) magnified image of the selected nanocrystal with measured interplanar distances, (c) 2D-FFT of the image with assigned Miller indices.

In the sample SnO_2_ nanocrystals were also present, with a TiO_2_-type primitive tetragonal structure (a = b = 4.745 Å, c = 3.260 Å, ICSD Col. Code – 219926) (Fig. 21).

#### 3.12.6 Determination of bandgap using Tauc method

**Fig. 22:**
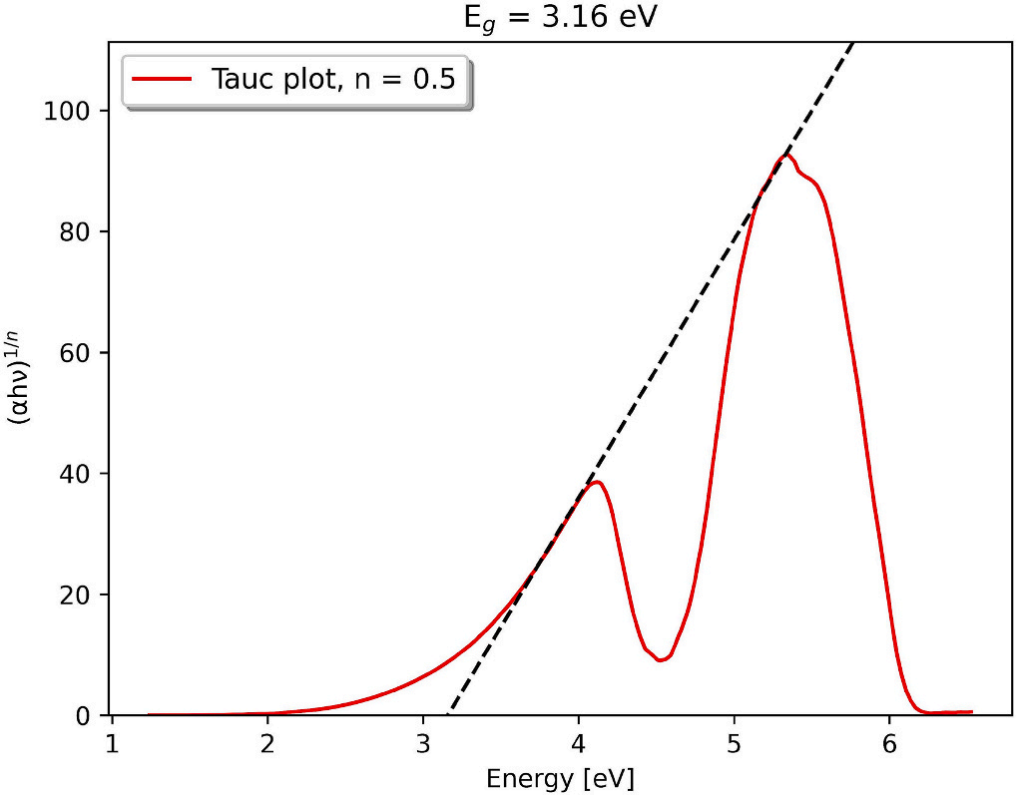
The Tauc plot for modified SnS catalyst after 5 cycles of sonocatalytic process (for direct allowed transition).

After 5 cycles of sonocatalytic process, the estimated value of bandgap for direct allowed transition increased to 3.16 eV from 2.16 eV (Fig. 22). Such significant increase is yet another evidence for the oxidation of SnS catalyst during the process as the bandgap of bulk SnO_2_ is 3.5 eV vs 1.3 eV for SnS [27], [80].

### 3.13 Kinetic modeling

Table 12 summarizes the results of fitting of various kinetic models to the experimental data resulting from catalytic experiments. In the case of photocatalytic experiments the best fitness was achieved with the pseudo-nth order kinetic model for each catalyst. The Langmuir-Hinshelwood (L-H) model fitted the data similarly like the pseudo-first order model despite the fact that the L-H model includes more parameters than the latter one (two vs one). In the case of SnS samples, when SnS was modified with 5 mg of Rhodamine B the order resulting from fitting with the pseudo-nth order model was 1.75. This value is significantly different when compared with those obtained for other catalysts. Taking into account that the pseudo-nth order kinetic model has two independent parameters (n, k) the near 0 order resulting from fitting is more a numerical issue and doesn’t have any real physicochemical meaning. The same is also with the fractional order of 1.75 for the photocatalytic process with SnS modified with 5 mg of RhB.

**Table 12:** Results of fitting of various kinetic models to data gathered from photocatalytic experiments.

Model	Pseudo-nth order			Pseudo-first order		Langmuir-Hinshelwood			
Catalyst	N	*k*, min^−1^∙(M)^1-n^	MAPE, %	*k*_1,_ min^−1^	MAPE, %	*K*, [l∙g^−1^_catalyst_]	*k*_2_, min^−1^	MAPE, %	
SnS (5 mg RhB)	1.75	0.143	2.9	0.00604	3.9	0.2425	0.0500	3.9	
SnS (10 mg RhB)	∼0	8.47E-05	3.8	0.00497	4.1	0.5000	0.0201	4.0	
SnS (15 mg RhB)	∼0	2.88E-05	6.8	0.00260	7.2	0.5000	0.0104	7.2	
SnS (20 mg RhB)	∼0	1.86E-06	2.4	0.000124	2.4	0.4999	0.00050	2.4	
SnS_2_ (5 mg RhB)	∼0	1.48E-05	2.7	0.000632	2.7	0.5000	0.00256	2.7	
SnS_2_ (10 mg RhB)	∼0	1.94E-05	0.9	0.000848	0.95	0.5000	0.00343	1.0	
SnS_2_ (15 mg RhB)	∼0	2.70E-05	0.8	0.00117	0.85	0.5000	0.00474	0.85	
SnS_2_ (20 mg RhB)	∼0	3.99E-05	2.5	0.00176	2.7	0.5000	0.00712	2.7	

Analogous data, but resulting from fitting to the data gathered during sonocatalytic processes, are presented in Table 13. The overall fitness of all models to the sonocatalytic data is lower than in the case of photocatalytic experiments which is due to the smaller number of experimental points. Additionally, during the sonocatalytic process the reaction temperature increases what affects the kinetic parameters and makes fitting more demanding. For the sonocatalytic process, there is no definitely the best model as it was in the case of the photocatalytic process.

**Table 13:** Results of fitting of various kinetic models to data gathered from sonocatalytic experiments.

Model	Pseudo-nth order			Pseudo-first order		Langmuir-Hinshelwood			
Catalyst	N	*k*, min^−1^∙M^1-n^	MAPE, %	*k*_1,_ min^−1^	MAPE, %	*K* [l∙g^−1^_catalyst_]	*k*_2_, min^−1^	MAPE, %	
SnS (5 mg of RhB)	1.59	1.34E-02	0.00	1.05E-02	9.26	150	4.14E-04	4.56	
SnS (10 mg of RhB)	∼0	5.94E-05	8.41	9.32E-03	16.62	150	4.09E-04	12.4	
SnS (15 mg of RhB)	∼0	1.04E-04	13.8	2.34E-13	9.09	4.14E-07	2.95E-07	9.09	
SnS (20 mg of RhB)	∼0	1.36E-04	1.15	2.11E-04	1.87	0.112	3.77E-03	1.87	
SnS_2_ (5 mg of RhB)	∼0	1.42E-04	9.68	1.93E-03	0.728	150	1.11E-04	0.57	
SnS_2_ (10 mg of RhB)	1.70	6.69E-11	9.09	1.79E-03	1.51	150	1.03E-04	1.37	
SnS_2_ (15 mg of RhB)	2.00	1.43E-02	1.87	1.89E-03	1.05	3.77E-03	1.00	1.05	
SnS_2_ (20 mg of RhB)	∼0	4.27E-05	0.523	2.33E-03	1.05	4.66E-03	1.00	1.05	

For the best photo- and sonocatalyst, also the modeling including the dependence of kinetic parameters on the amount of catalyst (c_cat_) was performed. The kinetic constant in the pseudo-first order reaction model was related with the concentration of catalyst according to the following equation:

(11) $$k_{1}=a∙{c_{\mathrm{cat}}}^{3}+b∙{c_{\mathrm{cat}}}^{2}+c∙c_{\mathrm{cat}}+d$$

Results of fitting are presented in Table 14, Table 15. For both processes the fitness of the Langmuir-Hinshelwood model and pseudo-first order kinetic model with linear dependence of kinetic parameter on catalyst’s concentration is quite similar according to the MAPE values. The pseudo-first kinetic model with both quadratic and cubic dependencies of kinetic parameter on catalyst’s concentration are much better fitted with quite similar MAPE values. This indicates that the assumption of quadratic dependence is enough to efficiently model the influence of catalyst’s concentration.

**Table 14:** Results of fitting of various kinetic models including the influence of catalyst amount to data gathered from photocatalytic experiments.

Model	MAPE [%]	*k*_1,_$l∙g^{-1}∙\min^{-1}$	*k*_-1_,$\min^{-1}$		*k*_2,_$\min^{-1}$
L-H	4.69	23.3	10.6		0.00447
k_1_ = f(c _cat_)	MAPE [%]	Polynomial coefficients			
		*a*$l^{3}\cdot g^{-3}\cdot \min^{-1}$	*b*$l^{2}\cdot g^{-2}\cdot \min^{-1}$	*c*$l\cdot g^{-1}\cdot \min^{-1}$	*d*$\min^{-1}$
k_1_ – linear	4.70			0.00944	3.47E-12
k_1_ – quadratic	1.95		0.0192	0.00203	6.46E-05
k_1_ – cubic	1.92	0.0152	0.00815	0.004042	1.96E-10

**Table 15:** Results of fitting of various kinetic models including the influence of catalyst amount to data gathered from sonocatalytic experiments.

Model	MAPE [%]	*k*_1,_$l∙g^{-1}∙\min^{-1}$	*k*_-1_,$\min^{-1}$		*k*_2,_$\min^{-1}$
L-H	12.4	2.66	0.951		0.00616
k_1_ = f(c _cat_)	MAPE [%]	Polynomial coefficients			
		*a*$l^{3}\cdot g^{-3}\cdot \min^{-1}$	*b*$l^{2}\cdot g^{-2}\cdot \min^{-1}$	*c*$l\cdot g^{-1}\cdot \min^{-1}$	*d*$\min^{-1}$
k_1_ – linear	12.7			0.01629	4.01E-11
k_1_ – quadratic	8.92		0.03815	2.67E-09	7.59E-04
k_1_ – cubic	8.79	1.80E-02	0.029374	4.55E-08	9.29E-04

## 4 Conclusions

We synthesized SnS and SnS_2_ quantum dots *in situ* modified with Rhodamine B using the sonochemical method. SEM observations showed the presence of nanoparticles in samples, while the EDX investigation proved the SnS or SnS_2_ elemental composition. XRD and Raman measurements confirmed the crystalline phases of SnS and SnS_2_ in the studied samples despite the strong fluorescence of Rhodamine B molecules. For this purpose, various laser lines were used to excite the sample, including resonant excitation (1064 nm) in Raman scattering for SnS. XPS analysis further evidenced the adsorption of Rhodamine B on the quantum dots and showed the binding of the RhB molecule to the Sn atom through Sn–C interactions. Investigation of optical band gaps based on the Tauc method revealed the quantum confinement effect in samples and the presence of additional transition corresponding to the Rhodamine B molecules. Modification of SnS quantum dots improved their photo- and sonocatalytic activity upon the modification with 5 mg of Rhodamine B added to the reaction mixture during synthesis. This improvement is linked with the increased wettability of catalyst as well as due to the improved absorption of light. The best catalyst (SnS quantum dots modified with 5 mg of Rhodamine B) allowed for 98 % and 62 % of color removal after 2 h of sonocatalytic and photocatalytic processes, respectively. Post-reaction XPS analysis of the best catalyst (SnS modified with 5 mg of RhB) provided crucial insights into its surface alterations during the degradation mechanism. The investigation highlighted a significant increase in carbon content (up to 60 at.%) accompanied by a pronounced dominance of C–C bonds (84 %) in the C 1 s spectrum, clearly indicating the strong adsorption of Metanil Yellow molecules or their organic degradation intermediates onto the surface of the quantum dots, forming a shielding layer. Concurrently, the energetic proximity of the O 1 s and Sn 3d core levels necessitated the assignment of combined components (O=C/O=Sn=O and SnS_2_/SnO_2_), successfully resolved by integrating structural data from XRD, SEM, and TEM investigations. Additionally, the disappearance of chlorine below the detection limit suggests the complete leaching or ion-exchange of Cl^-^ counterions from the Rhodamine B structure during the catalytic process. Scavenging experiments performed for this catalyst revealed that the most important reactive intermediate is the superoxide anion radical. Kinetic modeling showed that the best model for description of photocatalytic process was the pseudo-nth order kinetic model. For sonocatalytic process none of studied models (pseudo-nth order, pseudo-first order, and Langmuir-Hinshelwood model) was better than others. For both processes the best kinetic model to describe the influence of catalyst’s concentration on kinetic parameter was the pseudo-first order with quadratic dependence of that parameter on catalyst’s concentration. Although the catalyst underwent chemical oxidation during the sonocatalytic process, this study still demonstrates a strategy for improvement of catalytic activity. Further studies on the improvement of catalyst stability are thus required.

## CRediT authorship contribution statement

**Grzegorz Matyszczak:** Writing – review & editing, Writing – original draft, Visualization, Supervision, Methodology, Investigation, Formal analysis, Conceptualization. **Albert Yedzikhanau:** Visualization, Investigation, Formal analysis. **Tomasz Plocinski:** Visualization, Investigation, Formal analysis. **Piotr Dluzewski:** Visualization, Investigation, Formal analysis. **Aleksandra Fidler:** Writing – original draft, Visualization, Investigation, Formal analysis. **Cezariusz Jastrzebski:** Writing – original draft, Visualization, Investigation, Formal analysis. **Aleksandra Drzewiecka-Antonik:** Visualization, Investigation. **Anna Wolska:** Visualization, Investigation. **Jakub Zabrzycki:** Visualization, Investigation, Formal analysis. **Louis Peraud:** Visualization, Investigation. **Krzysztof Krawczyk:** Writing – review & editing, Supervision.

## Declaration of Generative AI and AI-assisted technologies in the writing process

Generative AI and AI-assisted technologies were not used in the writing process.

## Declaration of competing interest

The authors declare that they have no known competing financial interests or personal relationships that could have appeared to influence the work reported in this paper.
